# Day/Night Separation of Oxygenic Energy Metabolism and Nuclear DNA Replication in the Unicellular Red Alga *Cyanidioschyzon merolae*

**DOI:** 10.1128/mBio.00833-19

**Published:** 2019-07-02

**Authors:** Shin-ya Miyagishima, Atsuko Era, Tomohisa Hasunuma, Mami Matsuda, Shunsuke Hirooka, Nobuko Sumiya, Akihiko Kondo, Takayuki Fujiwara

**Affiliations:** a Department of Gene Function and Phenomics, National Institute of Genetics, Mishima, Shizuoka, Japan; b JST-Mirai Program, Japan Science and Technology Agency, Kawaguchi, Saitama, Japan; c Department of Genetics, Graduate University for Advanced Studies (SOKENDAI), Mishima, Shizuoka, Japan; d Graduate School of Science, Technology and Innovation, Kobe University, Nada, Kobe, Japan; e Engineering Biology Research Center, Kobe University, Nada, Kobe, Japan; f Biomass Engineering Program, RIKEN, Yokohama, Kanagawa, Japan; Dalhousie University; Max Planck Institute for Marine Microbiology

**Keywords:** cell cycle, *Cyanidioschyzon merolae*, endosymbiosis, photosynthetic oxidative stress

## Abstract

The transition from G_1_ to S phase and subsequent nuclear DNA replication in the cells of many species of eukaryotic algae occur predominantly during the evening and night in the absence of photosynthesis; however, little is known about how day/night changes in energy metabolism and cell cycle progression are coordinated and about the advantage conferred by the restriction of S phase to the night. Using a synchronous culture of the unicellular red alga Cyanidioschyzon merolae, we found that the levels of photosynthetic and respiratory activities peak during the morning and then decrease toward the evening and night, whereas the pathways for anaerobic consumption of pyruvate, produced by glycolysis, are upregulated during the evening and night as reported recently in the green alga Chlamydomonas reinhardtii. Inhibition of photosynthesis by 3-(3,4-dichlorophenyl)-1,1-dimethylurea (DCMU) largely reduced respiratory activity and the amplitude of the day/night rhythm of respiration, suggesting that the respiratory rhythm depends largely on photosynthetic activity. Even when the timing of G_1_/S-phase transition was uncoupled from the day/night rhythm by depletion of retinoblastoma-related (RBR) protein, the same patterns of photosynthesis and respiration were observed, suggesting that cell cycle progression and energy metabolism are regulated independently. Progression of the S phase under conditions of photosynthesis elevated the frequency of nuclear DNA double-strand breaks (DSB). These results suggest that the temporal separation of oxygenic energy metabolism, which causes oxidative stress, from nuclear DNA replication reduces the risk of DSB during cell proliferation in C. merolae.

## Abstract (precis)

Eukaryotes acquired chloroplasts through an endosymbiotic event in which a cyanobacterium or a unicellular eukaryotic alga was integrated into a previously nonphotosynthetic eukaryotic cell. Photosynthesis by chloroplasts enabled algae to expand their habitats and led to further evolution of land plants. However, photosynthesis causes greater oxidative stress than mitochondrion-based respiration. In seed plants, cell division is restricted to nonphotosynthetic meristematic tissues and populations of photosynthetic cells expand without cell division. Thus, seemingly, photosynthesis is spatially sequestrated from cell proliferation. In contrast, eukaryotic algae possess photosynthetic chloroplasts throughout their life cycle. Here we show that oxygenic energy conversion (daytime) and nuclear DNA replication (night time) are temporally sequestrated in C. merolae. This sequestration enables “safe” proliferation of cells and allows coexistence of chloroplasts and the eukaryotic host cell, as shown in yeast, where mitochondrial respiration and nuclear DNA replication are temporally sequestrated to reduce the mutation rate.

## INTRODUCTION

Photosynthesis, which is the primary route for the entry of energy into ecosystems, was developed in cyanobacteria around 3 billion years ago and was then introduced into eukaryotes >1 billion years ago by the establishment of chloroplasts through a cyanobacterial endosymbiotic event known as primary endosymbiosis. Chloroplasts then spread into several eukaryotic lineages by endosymbiotic events that incorporated unicellular eukaryotic algae into previously nonphotosynthetic eukaryotic cells (the secondary endosymbioses) (1).

Photosynthesis converts light energy into the chemical energy that directly supports the life of photosynthetic organisms and that indirectly supports the life of nonphotosynthetic organisms through food chains. However, electron transfer to oxygen from the respiratory chain or photosystems (O_2_^−^) or the excitation of oxygen by chlorophyll (^1^O_2_) produces reactive oxygen species (ROS), which damage various biomolecules. In addition, environmental stresses, such as heat, cold, drought, and light at high intensities, increase photosynthetic oxidative stress (2–4).

In seed plants, cell division is restricted to nonphotosynthetic meristematic tissues that possess nonphotosynthetic proplastids. Leaf cells with photosynthetic chloroplasts expand without any cell division (but with endoreduplication) (5). Thus, it appears that land plants spatially separate photosynthesis from cell proliferation and from the production of the next generation. In contrast to seed plants, cells of unicellular and multicellular eukaryotic algae, which emerged earlier than land plants based on the evolutionary perspective, possess chloroplasts throughout their life cycle. Thus, unlike seed plants, the proliferation of individual algal cells depends on photosynthesis, which causes oxidative stress.

In many lineages of eukaryotic algae of both primary and secondary endosymbiotic origin, cell division (resulting in an increase in cell number) occurs predominantly during a specific period in a day/night cycle (6–11). In most cases, cell division occurs at night. In some cases, nuclear DNA replication (S phase) was shown to occur in the evening and at night, during which time the cell cycle progression is regulated by a circadian rhythm (11–20).

It has been suggested that the restriction of some cellular events to night time by a circadian rhythm evolved because of cellular events that are hypersensitive to light irradiation (12, 21, 22). In the case of G_1_/S transition, regulation by a circadian rhythm was thought to protect DNA replication from UV damage on the basis of a study in the unicellular green alga Chlamydomonas reinhardtii (22), in which nuclear DNA is replicated during evening and early night (11, 17, 23, 24). In addition, in the case of species of green algae that possess cilia, including C. reinhardtii, it is assumed that the restriction of cell division to the night was a result of motile algae adapting to a situation in which cells must resorb their cilia prior to cell division in order to use their basal bodies to coordinate chromosome segregation and cytokinesis. Thus, the hypothesis assumes that, during the daytime, cilium-dependent phototaxis is required to optimize light absorption to maximize photosynthesis while reducing photosynthetic damage and that the period of cell division is hence delayed to the night as phototaxis is then not required (25).

Even though protection from UV and the constraints imposed by cilia (this is not applicable to algae without cilia) are important, the occurrence of cell division during the night, when photosynthesis does not occur, likely contributes to protection of nuclear DNA replication from photosynthetic oxidative stress. Supporting this assumption, mRNA levels of oxidative stress markers were found to be upregulated when the cycle progression was released from circadian restriction to the night in the unicellular nonciliated red alga C. merolae (18).

However, several questions in terms of the nature, mechanism, and biological significance of the restriction of the G_1_/S transition in algae still need to be addressed. (i) How is the energy for nuclear DNA replication and cell division supplied during the night when photosynthesis does not operate? Even without photosynthetic activity, respiration in mitochondria produces ROS, which is harmful if the cells undergo DNA replication. Regarding this point, during preparation of this paper, a study was published that showed that the unicellular green alga C. reinhardtii exhibits lower respiratory activity during the night than during the day whereas fermentation pathways are upregulated during the night (24). (ii) What relationships exist with respect to cell cycle progression and the respective forms of energy metabolism? (iii) Does the temporal sequestration of photosynthesis and nuclear DNA replication really contribute to “safe” cell proliferation?

To address these issues, we examined the day/night changes in photosynthetic and respiratory activities (transcriptome and metabolome) and the relationship between changes in energy metabolism and cell cycle progression in the unicellular red alga C. merolae. We found, in the current study, that mitochondrial respiration activity and anaerobic energy metabolism exhibit daily rhythms, as previously shown in C. reinhardtii (24), and that these rhythms largely depend on the daily changes in the photosynthetic activity. Oxygenic respiration is compromised in the evening and night, whereas anaerobic energy conversion is upregulated when cells undergo the G_1_/S transition. In addition, we found that the temporal separation of photosynthesis in the day and nuclear DNA replication in the evening/night reduces the risk of double-strand breaks (DSB).

## RESULTS

### Downregulation of photosynthesis and respiration during the evening and night in C. merolae and C. reinhardtii.

**FIG 1:**
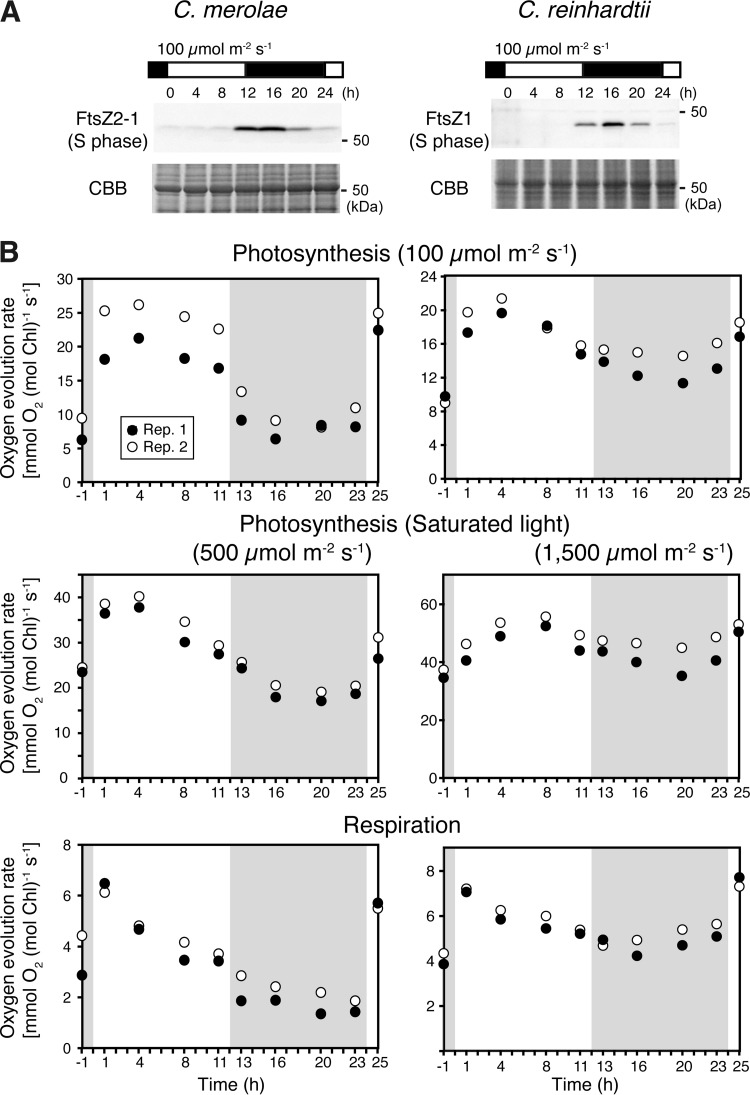
Temporal changes in photosynthetic and respiratory activities in synchronized cultures of the unicellular red alga C. merolae and unicellular green alga C. reinhardtii. (A) Immunoblot analysis showing the change in the level of FtsZ protein, which is expressed specifically during the S phase (26), in the synchronous cultures of C. merolae (FtsZ2-1) and C. reinhardtii (FtsZ1), where the onset of the second light period was defined as h 0. Coomassie brilliant blue (CBB) staining of the gel is shown as a loading control. (B) Temporal changes in respiratory and photosynthetic activities under light intensity of 100 μmol m^−2^ s^−1^ (the same intensity as the light period of the LD culture) and saturated light (500 μmol m^−2^ s^−1^ for C. merolae and 1,500 μmol m^−2^ s^−1^ for C. reinhardtii; the light saturation curves are shown in Fig. S1). Cells were cultured in an inorganic photoautotrophic medium and entrained by one round of an LD cycle before the measurements were carried out. The oxygen evolution rate of the culture under illumination (photosynthetic activity) and the oxygen consumption rate in darkness (respiratory activity) were measured in an electrode chamber immediately after sampling the culture at the indicated time points, where the onset of the second light period was defined as h 0. An aliquot of 10 mM NaHCO_3_ was added to the culture to measure the oxygen evolution of C. reinhardtii at 1,500 μmol m^−2^ s^−1^ only. The results of two independent cultures (Rep. 1 and Rep. 2) are shown.

To examine the relationship between day/night changes in energy metabolism and cell cycle progression in C. merolae, we cultured the cells in an inorganic photoautotrophic medium under a 12-h light/12-h dark (LD) cycle at a light intensity of 100 μmol m^−2^ s^−1^ during the light period. Synchronization of cell cycle progression was confirmed with immunoblotting, which showed that S-phase-specific FtsZ2-1 (26) was specifically expressed from h 12 to h 20, where the onset of the light period is defined as h 0 (Fig. 1A).

10.1128/mBio.00833-19.1 FIG S1 Light saturation curves of photosynthesis in C. merolae and C. reinhardtii. The cells were asynchronously cultured under continuous light (100 μmol m^−2^ s^−1^) and aerated with ordinary air (3 liters min^−1^). After sampling the log-phase culture, the oxygen evolution rate was measured at different light intensities at 40°C for C. merolae or 24°C for C. reinhardtii using a Clark-type oxygen electrode. For C. reinhardtii, the oxygen evolution rate present after the culture was supplemented with a 1/100 volume of 1 M NaHCO_3_ (so that oxygen evolution was not limited by the available carbon supply) was also measured. Because the medium for C. merolae was acidic (pH 2.5) and the addition of NaHCO_3_ yielded CO_2_ bubbles, which interfered the measurement, only values without NaHCO_3_ addition were measured in C. merolae. Data represent means ± standard deviations (SD) of results from three independent cultures. Download FIG S1, PDF file, 0.5 MB. Copyright © 2019 Miyagishima et al. 2019 Miyagishima et al. This content is distributed under the terms of the Creative Commons Attribution 4.0 International license.

Changes in photosynthetic and respiratory activities under LD cycle were examined by measuring the oxygen evolution rate under light conditions and consumption rate under dark conditions, respectively (Fig. 1B). The oxygen evolution rate was measured at 100 μmol m^−2^ s^−1^, which corresponded to the light intensity during the light period, and at 500 μmol m^−2^ s^−1^, which was above the light saturation point. The light saturation point of C. merolae was ∼400 μmol m^−2^ s^−1^ under our culture conditions (see Fig. S1 in the supplemental material). The photosynthetic activity of the culture at both 100 and 500 μmol m^−2^ s^−1^ peaked early in the light period (h 1 to 4) and then steadily decreased (Fig. 1B). Respiratory activity also exhibited a daily rhythm by reaching a maximum early in the light period (h 1) and then steadily decreasing (Fig. 1B).

To gain insights into the generality of the day/night rhythm of photosynthetic and respiratory activities in algae, we synchronized a culture of unicellular green alga C. reinhardtii in an inorganic photoautotrophic medium under conditions of an LD cycle with the light intensity of 100 μmol m^−2^ s^−1^ during the light period. The green and red algae diverged relatively soon after the emergence of primitive eukaryotic algae (27, 28). C. reinhardtii cells are known to enter the S/M phase predominantly during the evening and early part of the night (11, 17, 23). We confirmed synchronization of the cell cycle under our culture conditions with immunoblotting, which showed S-phase-specific FtsZ1 (26) to be expressed between h 12 and h 20 (Fig. 1A). Photosynthetic activity at 100 μmol m^−2^ s^−1^ and 1,500 μmol m^−2^ s^−1^, along with respiratory activity such as was very recently shown (24) in C. reinhardtii, exhibited a daily rhythm, as seen in C. merolae (Fig. 1B; the light saturation point of C. reinhardtii was ∼1,250 μmol m^−2^ s^−1^ under our culture conditions as shown in Fig. S1).

**FIG 2:**
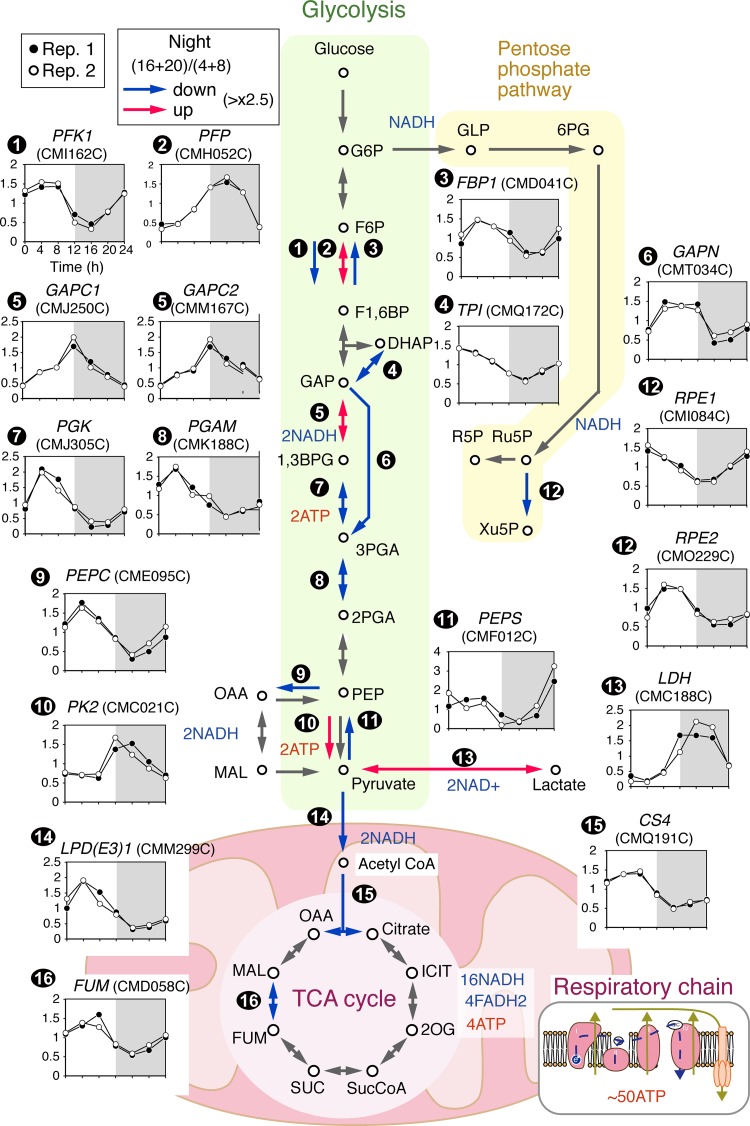
Temporal changes in the transcriptome related to the central metabolic pathway in a synchronized C. merolae culture. Cells were cultured in an inorganic photoautotrophic medium and subjected to an LD cycle. The transcriptome was examined by microarray analysis at the indicated time points (the onset of the second light period was defined as h 0). The respective amounts of mRNA relative to the total amount of mRNA were determined through quantile normalization. Microarray data representative of all the genes and certain selected genes are shown in Data Set S1. The results of two independent replicate cultures (Rep. 1 and Rep. 2; cultured at different times) are shown. Relative mRNA levels (*y* axis) at the indicated time points (*x* axis) are shown. The average concentration from h 0 to h 20 was normalized as 1.0 for the respective mRNAs. The determinations of the subcellular localization of the enzymes were based on previous analyses (32). The red and blue arrows indicate that mRNA was upregulated ([sum of the levels measured at h 16 and h 20/sum of the levels measured at h 4 and h 8] > 2.5) and downregulated ([sum of the levels measured at h 16 and h 20/sum of the levels measured at h 4 and h 8] < 0.4) during the night, respectively. The gray arrow indicates that the magnitude of change in the mRNA level was lower than that determined as described above.

To understand how the downregulation of both photosynthesis and respiration during the evening and night affects the metabolic state of C. merolae cells, we examined the day/night changes in the transcriptome by microarray analyses (the relative levels per total mRNA at each time point are shown in Fig. 2; see also Fig. S2 and Fig. S3 and Data Set S1 in the supplemental material).

10.1128/mBio.00833-19.2 FIG S2 Temporal changes in the transcriptome related to the photosystems, CO_2_ fixation (Calvin–Benson–Bassham cycle), respiratory chain, and cell cycle progression in a synchronized C. merolae culture. Results were obtained as described in the Fig. 2 legend. Microarray data from all the genes and from examinations of certain selected genes are shown in Data Set S1. Results from two independent cultures (Rep. 1 and Rep. 2; cultured at different times) are shown. The color scale at the top right represents the fold change in mRNA abundance. In the diagrams, the respective components are colored according to the ratio of the night data (sum of h 16 and h 20) to the day data (sum of h 4 and h 8). Organelle-genome-encoded components indicated in gray were not examined. In the color bar showing the temporal change, the data representing the average of the values measured from h 0 to h 20 were assigned a value of 1.0; the relative levels measured at the respective time points are shown in the color scale. The data representing the protein composition in the photosystems (70) and the subcellular localization of the enzymes involved in carbohydrate metabolism (32) are based on previous analyses. The protein composition in the respiratory chain is based on data from the Kyoto Encyclopedia of Genes and Genomes (KEGG) database. Download FIG S2, PDF file, 1.3 MB. Copyright © 2019 Miyagishima et al. 2019 Miyagishima et al. This content is distributed under the terms of the Creative Commons Attribution 4.0 International license.

10.1128/mBio.00833-19.3 FIG S3 Temporal changes in the mRNA levels of genes encoding pyruvate kinases, components of the pyruvate dehydrogenase complex, and TCA cycle enzymes in a synchronized C. merolae culture. The details are described in the Fig. 2 legend. Download FIG S3, PDF file, 0.4 MB. Copyright © 2019 Miyagishima et al. 2019 Miyagishima et al. This content is distributed under the terms of the Creative Commons Attribution 4.0 International license.

10.1128/mBio.00833-19.10 DATA SET S1 Transcriptome data and primers used in this study. Download Data Set S1, XLSX file, 2.4 MB. Copyright © 2019 Miyagishima et al. 2019 Miyagishima et al. This content is distributed under the terms of the Creative Commons Attribution 4.0 International license.

Consistent with the daily change in photosynthetic activity (Fig. 1B), nuclear genes encoding components of chloroplast photosystems and Calvin-Benson-Bassham cycle enzymes were downregulated during the night (Fig. S2), as observed in other algae and plants (23, 29, 30). In addition, cell cycle-related genes exhibited a clear day/night rhythm. The mRNAs of S-phase-specific and M-phase-specific genes accumulated specifically during the night (Fig. S2), and this is consistent with the restriction of G_1_/S transition to the night in C. merolae (Fig. 1A) (18, 31).

The nuclear genes encoding components of the mitochondrial respiratory chain exhibited time course expression patterns that were different from those seen with nuclear genes encoding components of the photosystem. Some were upregulated or downregulated during the night, whereas others exhibited marginal changes over a 24-h period (Fig. S2). The mitochondrial tricarboxylic acid (TCA) cycle provides electrons (via NADH) to the respiratory chain. Most of the mRNAs that encode enzymes of the TCA cycle exhibited marginal changes (Fig. 2; see also Fig. S3). However, we found that mRNAs encoding enzymes that are involved in the entrance into the TCA cycle, namely, pyruvate dehydrogenase and citrate synthase, were downregulated during the night (Fig. 2; see also Fig. S3). The pyruvate dehydrogenase complex connects glycolysis with the TCA cycle. Among the genes encoding components of the pyruvate dehydrogenase complex, for which mitochondrial localization was confirmed previously (32), the day to night differences in levels of E1 alpha, E1 beta, and E2 mRNA were less than 2.5-fold (Fig. S3). In contrast, the level of E3 (dihydrolipoamide dehydrogenase; LPD) mRNA exhibited an ∼5-fold variation within a 24-h period, reaching a maximum at h 4 and a minimum at h 16 (Fig. 2, step 14; see also Fig. S3). Mitochondrial citrate synthase catalyzes the reaction between acetyl-coenzyme A (acetyl-CoA) and oxaloacetic acid to form citric acid, which is a rate-determining step in the TCA cycle (33, 34). Similarly to E3 of the pyruvate dehydrogenase complex, the level of citrate synthase (CS4) mRNA, for which mitochondrial localization was confirmed previously (32), exhibited a day/night rhythm, reaching a maximum between h 4 and h 8 and a minimum at h 16 (Fig. 2, step 15).

Next, we examined day/night changes in levels of mRNAs that are involved in cytosolic glycolysis, which supplies pyruvate to mitochondrial pyruvate dehydrogenase in order to mediate the operation of the TCA cycle (Fig. 2). Cytosolic localization of the respective proteins involved in cytosolic glycolysis was confirmed previously (32). Pyruvate kinase (PK) transfers a phosphoryl group from phosphoenolpyruvate (PEP) to ADP to produce pyruvate and ATP; this is the rate-limiting reaction in glycolysis (33, 34). The level of cytosolic pyruvate kinase (PK2) mRNA exhibited a day/night rhythm in which the level was higher during the night than during the day (Fig. 2, step 10). In contrast, the level of mRNA encoding PEP synthetase (EC 2.7.9.2), which converts pyruvate to phosphoenolpyruvate by consuming ATP, exhibited a day/night rhythm that was opposite that seen with PK2 (Fig. 2, step 11). In summary, the mRNA for cytosolic pyruvate and ATP production (pyruvate kinase) was upregulated during the night, whereas those related to the consumption of pyruvate by the mitochondrial TCA cycle (pyruvate dehydrogenase and citrate synthase) were downregulated during the night.

The observations of the day/night change in the respiratory activity and the results of transcriptome analysis suggest that, during the night, cytosolic pyruvate is probably processed by metabolic pathways other than the mitochondrial TCA cycle. In algae, pyruvate is processed by various fermentation pathways in which ethanol, formate, acetate, and lactate are produced anaerobically from pyruvate and excreted from cells, especially under anaerobic conditions (35, 36). C. merolae contains both lactate and alcohol dehydrogenases but lacks formate and acetate fermentation pathways (37).

l-Lactate dehydrogenase (LDH) catalyzes the reversible oxidation of lactate to pyruvate using NAD^+^. The reverse reaction (pyruvate and NADH to lactate and NAD^+^) is favored at a physiological pH value. One of its primary purposes is to replenish the pool of NAD^+^ under conditions of insufficient oxygen to allow glycolysis and the production of ATP to continue (33, 34). The C. merolae genome carries five genes encoding LDH proteins (CMA145C, CMC188C, CMI306C, CMJ002C, and CMK006C). The cDNA sequences of these five *orf* genes are almost (>98%) identical to one another, and the transcripts are indistinguishable in microarray analyses. Therefore, in this study, all five genes were equated and represented as CMC188C. The results of the microarray analysis indicated that the level of CMC188C mRNA exhibited a day/night rhythm and that the level was higher in the night than in the day (Fig. 2, step 13). In summary, mRNAs related to pyruvate consumption by the TCA cycle for aerobic respiration in the mitochondria were downregulated whereas those for glycolysis and lactate fermentation were upregulated during the night.

**FIG 3:**
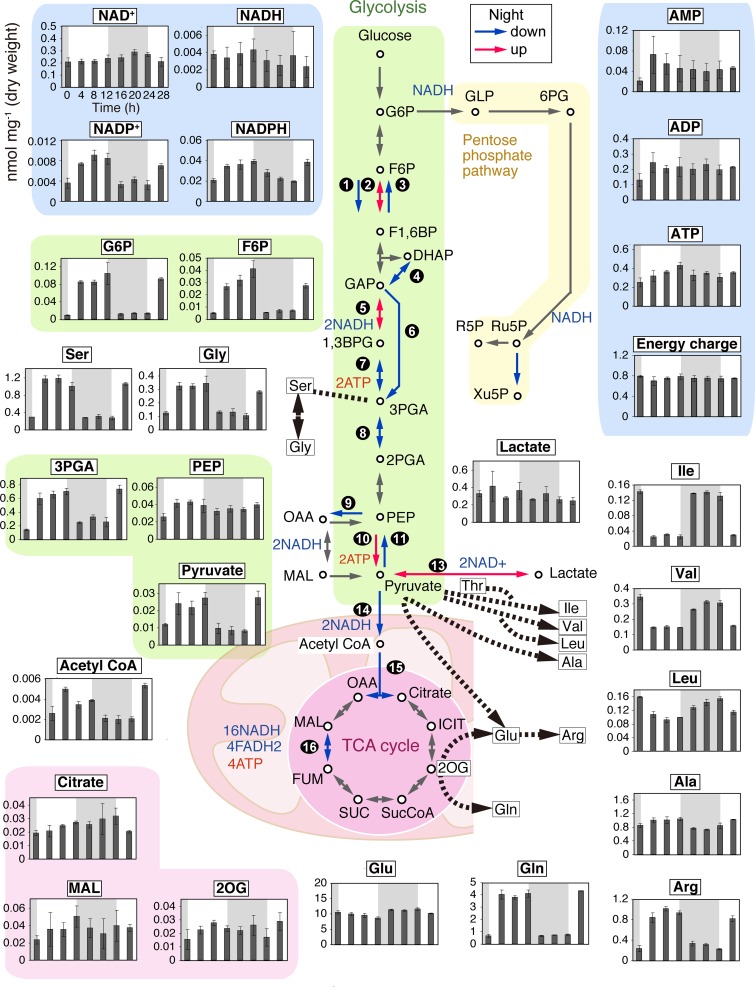
Temporal changes in cellular metabolite levels related to the central metabolic pathway in a synchronized C. merolae culture. Cells were cultured in an inorganic photoautotrophic medium and subjected to an LD cycle. The metabolome at indicated time points (the onset of the second light period was defined as h 0) was examined by CE-MS. The metabolite concentration (*y* axis; nanomoles/milligram cellular dry weight), except for the energy charge, is shown at indicated time points (*x* axis). The energy charge was calculated according to the following equation: ([ATP]+0.5[ADP])/([ATP]+[ADP]+[AMP]) (69). Each data point represents means ± standard deviations (SD) of results from three independent cultures. The red and blue arrows in the diagram indicate that the mRNA encoding the corresponding enzyme was upregulated and downregulated, respectively, during the night as described in the Fig. 2 legend.

Changes in the transcriptome do not always correlate with changes in associated metabolic activities, because many enzymes are regulated not only by transcriptional adjustments but also by posttranslational modifications. The downregulation of mRNAs encoding pyruvate dehydrogenase and citrate synthase during the evening and night seemingly corresponds to the day/night change in respiratory activity. To examine whether pyruvate is processed mainly by anaerobic metabolic pathways rather than by mitochondrial respiration, as suggested by the transcriptome change, we examined intracellular metabolome changes under LD conditions using capillary electrophoresis-mass spectrometry (CE-MS) (the values per cellular dry weight at respective time points are shown in Fig. 3; see also Fig. S4).

10.1128/mBio.00833-19.4 FIG S4 Data representing temporal changes in metabolite levels in a synchronized C. merolae culture that were not included in Fig. 3. The methodological details are described in the Fig. 3 legend. Download FIG S4, PDF file, 0.5 MB. Copyright © 2019 Miyagishima et al. 2019 Miyagishima et al. This content is distributed under the terms of the Creative Commons Attribution 4.0 International license.

The intracellular pyruvate concentration was shown to increase during the day and decrease during the night (Fig. 3). In contrast, intracellular lactate was maintained at an almost constant concentration during the course of the day and night (Fig. 3). These results likely reflect that the rate of lactate synthesis per unit of substrate (pyruvate) increase during the night. In addition, CE-MS analyses showed that the acetyl-CoA concentration decreased whereas some amino acids (leucine, isoleucine, valine, and lysine) whose production consumes pyruvate (33) accumulated during the night (Fig. 3; see also Fig. S4). These results suggest that pyruvate is mainly processed anaerobically during the night in C. merolae.

Additionally, changes in the concentrations (per cellular dry weight) of intracellular ATP and the reductants NADPH and NADH were shown to be less than 2-fold over a 28 h period under LD conditions (Fig. 3). On the basis of the adenylate kinase reaction (2ADP ↔ ATP + AMP), the energy charge, which takes into account the concentrations of ATP, ADP, and AMP, is used as an index of cellular energy status (38). The energy charge exhibited an almost constant value throughout the LD cycle (Fig. 3) as calculated by the following formula (38):

([ATP]+0.5[ADP])/([ATP]+[ADP]+[AMP])

These results suggest that the energy state is maintained in C. merolae cells during the LD cycle, even though photosynthesis ceases and respiratory activity is compromised during the dark period.

Additionally, using high-performance liquid chromatography, we attempted to quantify the lactate concentration of C. merolae culture medium with or without aeration, but the concentrations were below the detection limit, as was the situation in C. reinhardtii wild-type cells, in which the extracellular lactate concentrations, but not the intracellular lactate concentrations, were below the detection limit under both aerobic and anaerobic conditions (39).

### Relationships between daily rhythms of energy metabolism and cell cycle progression.

**FIG 4:**
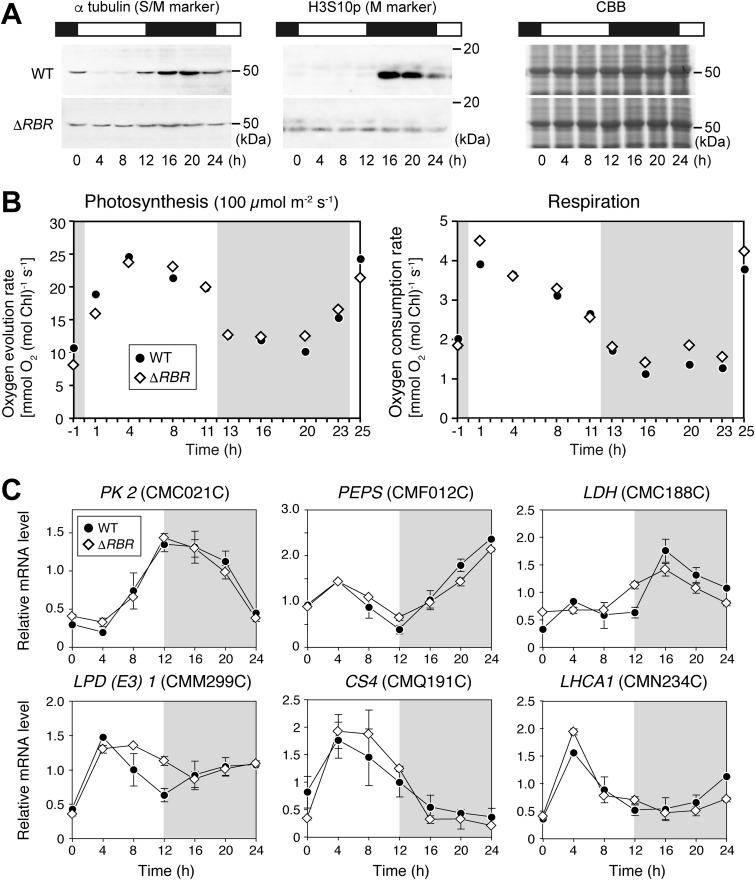
The relationship between the cell cycle and temporal changes in photosynthetic activity and respiratory activity in a synchronized C. merolae culture. (A) Immunoblot analysis data, showing changes in levels of alpha-tubulin, which is expressed specifically during the S and M phases in C. merolae (43), and histone H3 phosphorylated at serine 10 (H3S10p; an M-phase marker) during an LD cycle in the wild-type (WT) and *ΔRBR* strains (the onset of the second light period is defined as h 0). CBB staining of the gel is shown as a loading control. (B) Oxygen evolution rate in the light (100 μmol m^−2^ s^−1^) (photosynthetic activity under conditions of light intensity matching that seen in the light period of the LD culture) and oxygen consumption rate in the dark (respiratory activity) of the culture were measured immediately after sampling the culture at the indicated time points (the onset of the second light period was defined as h 0). Data representing photosynthetic activity under saturated light (500 μmol m^−2^ s^−1^) are shown in Fig. S6. (C) Quantitative RT-PCR analyses showing the temporal changes in concentrations of mRNA encoding a light-harvesting chlorophyll-binding (LHCA1) protein and enzymes related to pyruvate metabolism in the WT and *ΔRBR* strains. mRNA encoding dynamin-related protein 3 (*DRP3*; CME019C) was used as the internal control. Each data point represents means ± SD of results from three biological replicates. The average mRNA level from h 0 to h 20 in the respective strains was defined as 1.0. The results determined for another independent *ΔRBR* clone are shown in Fig. S5.

To investigate whether there is any causal relationship(s) between the day/night rhythms of energy metabolism and cell cycle progression, we examined the effects of retinoblastoma-related (RBR) protein depletion on cell cycle progression, day/night changes in photosynthetic and respiratory activities, and mRNA levels of genes associated with pyruvate metabolism and photosynthesis (a comparison between the wild-type strain and Δ*RBR* clone 1 is shown in Fig. 4; a comparison between the wild-type strain and Δ*RBR* clone 2 is shown in Fig. S5).

10.1128/mBio.00833-19.5 FIG S5 Comparison between the wild-type strain and Δ*RBR* clone 2 (clone independent from clone 1 shown in Fig. 4). The details are described in the Fig. 4 legend. Download FIG S5, PDF file, 0.8 MB. Copyright © 2019 Miyagishima et al. 2019 Miyagishima et al. This content is distributed under the terms of the Creative Commons Attribution 4.0 International license.

10.1128/mBio.00833-19.6 FIG S6 Change in photosynthetic activity under saturated light in the wild-type and Δ*RBR* strains under conditions of the LD cycle. Oxygen evolution rate at 500 μmol m^−2^ s^−1^ was measured immediately after sampling the culture at the indicated time points (the onset of the second light period was defined as h 0). Data from two independent Δ*RBR* clones (clones 1 and 2) are shown. The culture and measurements of the Δ*RBR* clones were performed at the same time as those performed for the wild-type strain (Fig. 1B; 500 μmol m^−2^ s^−1^). The same results (Fig. 1B; 500 μmol m^−2^ s^−1^) are shown for comparison. The details are described in the Fig. 4 legend. Download FIG S6, PDF file, 0.4 MB. Copyright © 2019 Miyagishima et al. 2019 Miyagishima et al. This content is distributed under the terms of the Creative Commons Attribution 4.0 International license.

RBR is an inhibitor of the G_1_/S transition widely conserved in eukaryotes, including algae. RBR represses the G_1_/S transition by binding the E2F-DP transcription factor heterodimer, which binds certain specific *cis* elements of S-phase genes, such as those encoding cyclin A and DNA polymerases (13, 18, 40–42). During the G_1_ phase, the G_1_ cyclin concentration increases in concert with cellular growth, and a G_1_ cyclin–cyclin-dependent kinase (CDK) complex phosphorylates the RBR. This phosphorylation inactivates RBR, which then activates the transcription of the S-phase genes by E2F-DP (13, 18, 40–42). We previously showed that the RBR–E2F-DP pathway is involved in the day/night regulation of the G_1_/S transition in C. merolae and that the RBR depletion abolished the restriction of the G_1_/S transition to the night (18). As shown previously (18), after entrainment by LD, the Δ*RBR* culture contained a certain population of S/M-phase cells throughout the LD cycle, unlike the wild-type culture, in which S/M-phase cells were specifically detected during the night (Fig. 4A; see also Fig. S5A). Note that alpha-tubulin and beta-tubulin proteins are expressed specifically during the S and M phases and that histone H3 is phosphorylated at serine 10 (H3S10p) specifically during the M phase in C. merolae (43).

Even though cell cycle progression was uncoupled from day/night rhythms, the photosynthetic activity (at both 100 and 500 μmol m^−2^ S^−1^) and the respiratory activity of the Δ*RBR* culture exhibited a day/night rhythm almost identical to that exhibited by the wild-type culture (Fig. 4B; see also Fig. S5B and Fig. S6). Quantitative reverse transcription-PCR (RT-PCR) analyses showed that the Δ*RBR* strain exhibited day/night rhythms of mRNAs encoding cytosolic pyruvate kinase (*PK2*), PEP synthase (*PEPS*), mitochondrial pyruvate decarboxylase E3 [*LPD* (*E3*)], citrate synthase (*CS4*), and the chlorophyll *a*-binding protein (*LHCA1*; one of two *LHCA* genes carried in the C. merolae genome) comparable to the day/night rhythms exhibited by the wild-type strain (Fig. 4C; see also Fig. S5C). Although *LDH* exhibited rhythms that differed slightly between the wild-type strain and two independent Δ*RBR* clones, the mRNA levels peaked during the day in all of the strains (Fig. 4C; see also Fig. S5C).

The observations detailed above showed that the day/night rhythms in photosynthetic activity and respiratory activity and in the associated mRNA levels were not markedly affected when cell cycle progression was uncoupled from the day/night rhythm (Δ*RBR*). Thus, it is suggested that energy metabolism and cell cycle progression are independently regulated by day/night rhythms in C. merolae.

### Relationship between day/night rhythms of photosynthesis and respiration.

**FIG 5:**
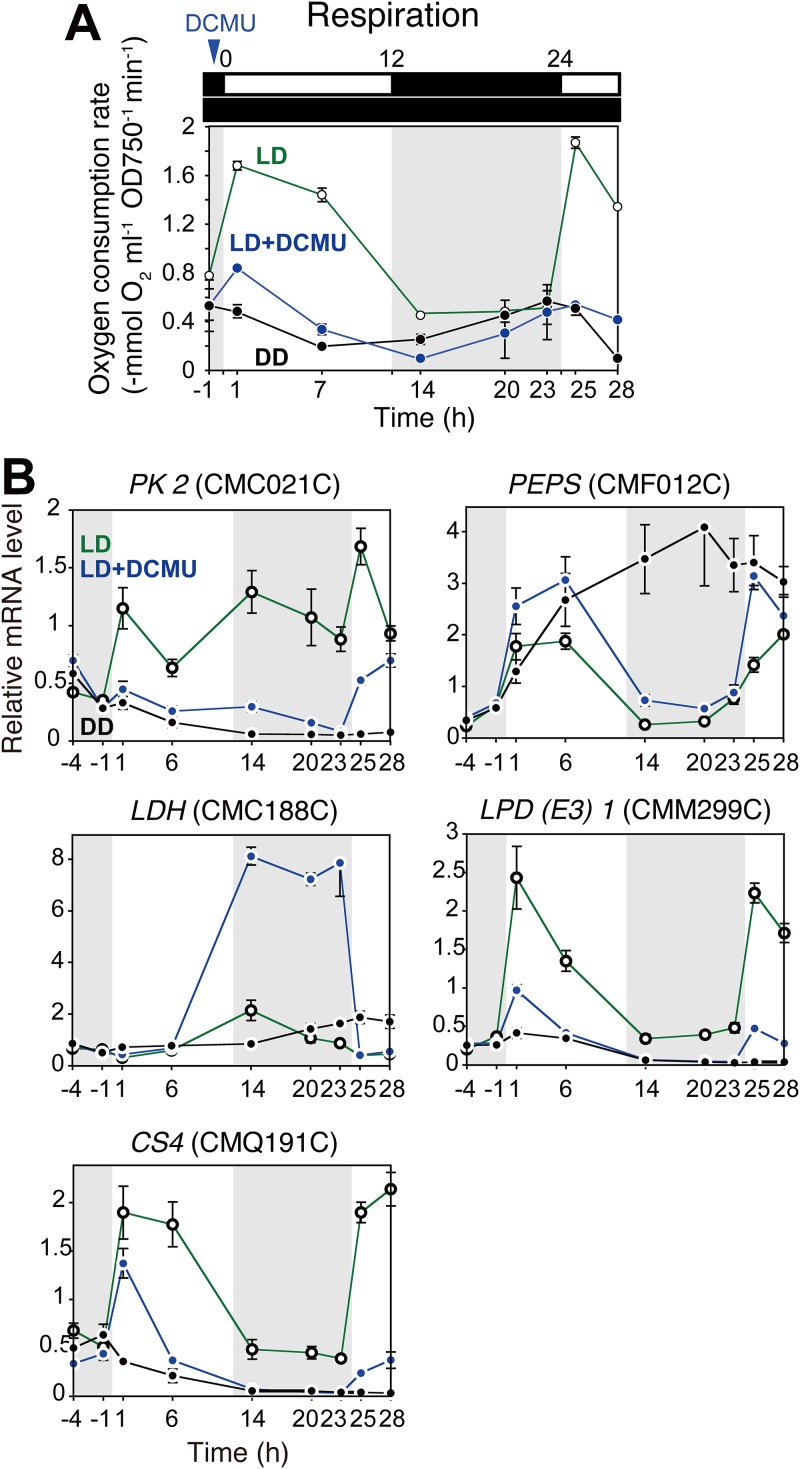
The relationship between light and photosynthesis and temporal changes in respiratory activities in C. merolae. Cells were cultured in three bottles of inorganic photoautotrophic medium and entrained by one round of an LD cycle. Next, two bottles were cultured under LD. A 1/5,000 volume of 50 mM DCMU dissolved in ethanol (LD+DCMU) or ethanol alone (LD) was added to the culture 30 min before h 0 (the onset of the second light period was defined as h 0). Another bottle was cultured under continuous dark (DD) after the entrainment. (A) The oxygen consumption rate of the culture in the dark (respiratory activity) was measured immediately after sampling the culture at indicated time points. Results from one experiment are shown, and results from another replicate cultured at a different time are shown in Fig. S7. (B) Quantitative RT-PCR analyses showing temporal changes in levels of mRNAs encoding enzymes related to pyruvate metabolism in LD, LD+DCMU, and DD cultures. *DRP3* (CME019C) was used as the internal control. The data points represent means ± SD of results from three independent cultures. The average mRNA concentration from h 1 to h 23 in the LD culture was defined as 1.0.

In land plants and algae, photosynthetic activity in the chloroplast accelerates respiratory activity in the mitochondrion (44–48). Thus, it is likely that the day/night rhythm of respiration observed in C. merolae and C. reinhardtii (Fig. 1) is caused by the day/night rhythm of photosynthetic activity. To test this hypothesis, we examined the effect of inhibiting photosynthesis on the rhythms of respiratory activity and relevant gene expression. To this end, a C. merolae culture was synchronized by one round of LD and then either kept under LD conditions, with or without 3-(3,4-dichlorophenyl)-1,1-dimethylurea (DCMU) (an inhibitor of photosynthetic electron flow), or cultured under conditions of continuous dark (DD; Fig. 5).

10.1128/mBio.00833-19.7 FIG S7 Results of analysis of a biological replicate (cultured at different times) of the experiment described in the Fig. 5A legend. Download FIG S7, PDF file, 0.4 MB. Copyright © 2019 Miyagishima et al. 2019 Miyagishima et al. This content is distributed under the terms of the Creative Commons Attribution 4.0 International license.

In the control culture under LD conditions in the absence of DCMU, respiratory activity increased immediately upon illumination (from h −1 to h 1 and from h 23 to h 25). In contrast, under DD conditions, the day/night rhythm of respiratory activity was nearly abolished (Fig. 5A; see also Fig. S7). Consistent with this observation, quantitative RT-PCR analyses showed that pyruvate-related mRNA levels did not exhibit ∼24-h rhythms under DD conditions, which is in contrast to the outcome seen under LD conditions (Fig. 5B). The relative levels of mRNAs of cytosolic *PK2*, mitochondrial *LPD* (*E3*), and *CS4* decreased under DD conditions whereas those of *LDH* and *PEPS* mRNAs increased (Fig. 5B).

When DCMU was added 0.5 h before the onset of LD (h 0), the levels of respiratory activity and the amplitudes of the day/night rhythm of respiration were largely suppressed compared with those seen with the control culture without DCMU (Fig. 5A; see also Fig. S7 [LD]). However, even in the presence of DCMU, the level of respiratory activity still increased slightly upon illumination (from h −1 to h 1) and then continued to decrease until the evening (from h 1 to h 14), as in the LD culture. After that, respiratory activity in the presence of DCMU again increased slightly toward the following morning (h 14 to 25) and then decreased as seen in the control culture (Fig. 5A; see also Fig. S7). Consistent with this observation, patterns of temporal changes in pyruvate-related mRNA levels in the presence of DCMU were similar to those in the control LD culture (in the absence of DCMU); however, the levels and amplitudes of *PK2*, *LPD* (*E3*), and *CS4* decreased in comparison with the results seen with the control culture, except in the case of *LDH* (Fig. 5B). The level of *LDH* mRNA increased under conditions of DCMU treatment during the night (Fig. 5B), although the reason for this rise was unclear.

These results suggest that (i) C. merolae generates low amplitudes of day/night rhythms of gene expression and respiratory activity, depending on dark-light shift, even without photosynthesis under LD conditions, and that (ii) photosynthetic activity increases the levels of gene expression and respiratory activity and thus the magnitude of their day/night rhythms.

Regarding point i above, DCMU inhibits the linear electron flow from photosystem II to photosystem I for NADPH production and thus prevents cellular growth. However, even in the presence of DCMU, the cyclic electron flow around photosystem I produces ATP under conditions of exposure to light (49). Thus, the upregulation of respiratory activity and the level of gene expression observed upon illumination in the presence of DCMU were caused by light stimulation or cyclic electron flow in the chloroplast.

### Temporal restrictions of oxygenic energy metabolism in the day and nuclear DNA replication in the night reduce the risk of nuclear DNA DSB.

The current study showed that photosynthetic and respiratory activities are at their highest early in the morning and decrease toward the evening when cells enter the S phase in C. merolae and C. reinhardtii. It was shown previously that DNA in the S phase was the most susceptible to oxidative stress in mammalian cells (50). On the basis of these results, it is plausible that the temporal sequestration of nuclear DNA replication from ROS-producing photosynthetic and respiratory activities is likely to ensure the relatively safe proliferation of cells.

**FIG 6:**
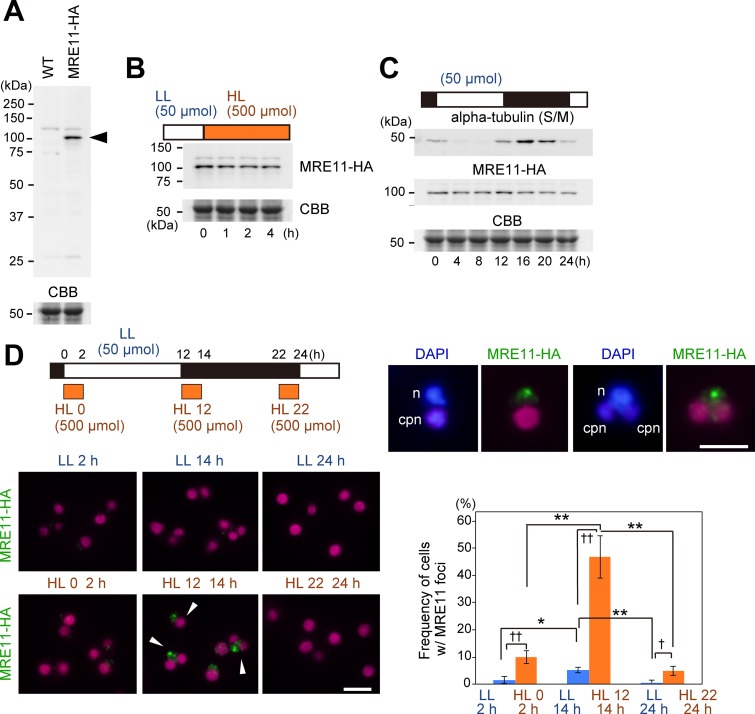
Change in susceptibility to nuclear DNA damage during cell cycle progression in C. merolae. (A) An immunoblot analysis showing the expression of C-terminal 3× HA-tagged MRE11 in the MRE11-HA strain. The 3× HA-encoding sequence was integrated just before the stop codon of *MRE11 orf* in the chromosome to express the HA-tagged protein by the *MRE11* promoter. The wild-type (WT) strain was used as a negative control. The MRE11-HA protein was detected with the anti-HA antibody. (B) Immunoblot analysis showing the change in MRE11-HA level, when an asynchronous culture was transferred from low-light conditions (LL; 50 μmol m^−2^ s^−1^) to high-light conditions (HL; 500 μmol m^−2^ s^−1^) to increase photosynthetic oxidative stresses. (C) Immunoblot analysis showing the change in the MRE11-HA protein concentration during cell cycle progression. MRE11-HA cells were synchronized by the use of an LD cycle, and the protein level during the second LD cycle was examined (the onset of the second light period was defined as h 0). The level of alpha-tubulin, which is expressed specifically during the S and M phases in C. merolae (43), is also shown. CBB staining of the gel is shown as a loading control in panels A to C. (D) Immunofluorescence images showing the formation of MRE11-HA foci in the nuclei under conditions of high levels of light stress. MRE11-HA cells were synchronized by the use of a low-light LD cycle (50 μmol m^−2^ s^−1^). Cultures were kept in a low-light LD cycle (LL) or exposed to a high-light cycle (500 μmol m^−2^ s^−1^) for 2 h from h 0 (HL 0) or h 12 (HL 12) or h 22 (HL 22) during the second LD cycle to increase photosynthetic oxidative stresses. Images obtained by immunofluorescence microscopy, showing MRE11-HA protein localization at h 2 for LL and HL 0, h 14 for LL and HL 12, and h 24 for LL and HL 22. Two representative cells with MRE11-HA foci (HL 12 at h 14) with DAPI staining are also shown. Green, MRE11-HA detected with the HA antibody; blue, DNA stained with DAPI; magenta, autofluorescence of the chloroplast; *n*, nucleus; *cpn*, chloroplast nucleoid. The percentage of cells that exhibited MRE11 foci in the nuclei (examples are indicated with arrowheads in microscopic images) in total cells is indicated on the graph. Each data point represents means ± SD of results from three biological replicates (*n *>* *200 cells for each sample). Asterisks denote statistically significant differences (*, *P < *0.05; **, *P < *0.01 [Tukey's test]; †, *P < *0.05; ††, *P < *0.01 [*t* test]). Scale bars = 5 μm.

To test this possibility, we examined the possibly toxic effect of the simultaneous occurrence of photosynthesis, which also increases respiratory activity (Fig. 5), and nuclear DNA replication. To assess the potential toxicity, we determined the frequency of DSB in nuclear DNA based on the formation of MRE11 foci in the nuclei. The MRE11 protein is well conserved in eukaryotes and is involved in DSB repair by forming a focus with RAD50 and NBS1 in the nucleus (51). To visualize MRE11 focus formation in C. merolae by immunofluorescence microscopy, a 3× hemagglutinin (HA)-coding sequence was inserted into the chromosomal *MRE11* locus just before the stop codon to express the C-terminal 3× HA fusion protein by the endogenous *MRE11* promoter (Fig. 6A).

Immunoblotting with an anti-HA antibody showed that total cellular MRE11-HA was maintained at an almost constant level following a shift to high light intensity (50 to 500 μmol m^−2^ s^−1^; Fig. 6B). The exposure of cells to conditions of high levels of light is known to cause raised levels of photosynthetic oxidative stress. The elevation of chlorophyll excitation generates excessive electron flow in the photosystem that cannot be consumed by CO_2_ reduction, which in turn escalates the rate of electron transfer to oxygen molecules and ultimately generates ROS (2–4). In addition, immunoblot analysis showed that the MRE11-HA level was almost constant during the LD cycle and thus throughout the progression of the cell cycle (Fig. 6C).

When the culture that was synchronized under LD conditions (50 μmol m^−2^ s^−1^ during the light period) was exposed to a high level of light (500 μmol m^−2^ s^−1^) for 2 h from h 0, 12, or 22, MRE11-HA foci were observed in the nuclei by immunofluorescence microscopy with an anti-HA antibody, especially in cells exposed to high levels of light intensity from h 12 to h 14 (Fig. 6D). The incidence of cells containing MRE11-HA foci was about five times higher in the culture exposed to high light from h 12 to h 14, which corresponded with the S phase, than in the culture exposed to high light either from h 0 to h 2 or from h 22 to h 24, which corresponded to the G_1_ phase (Fig. 6D). Even in the control culture under LD conditions without exposure to high light, the frequency of cells containing MRE11-HA foci was higher at h 14 (S phase) than at h 2 or h 24 (G_1_ phase) (Fig. 6D). These results suggest that S-phase cells are more susceptible to DNA damage caused by light and/or photosynthetic oxidative stress.

**FIG 7:**
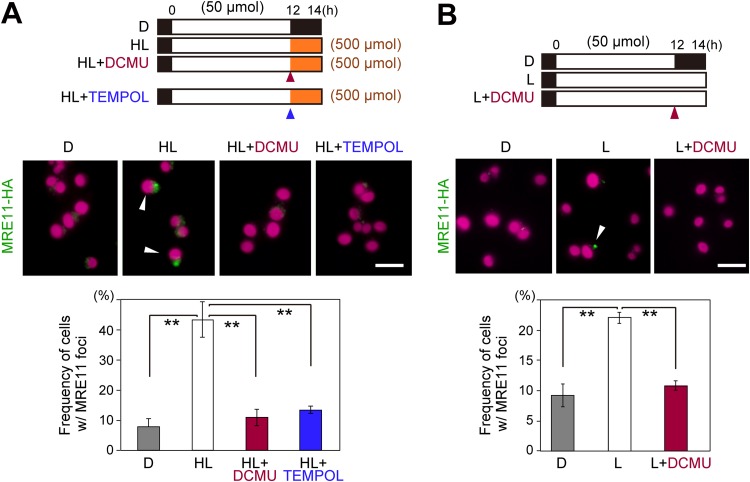
Effect of photosynthesis on cellular susceptibility to nuclear DNA damage in C. merolae. (A) MRE11-HA cells were synchronized by the use of a low-light LD cycle (50 μmol m^−2^ s^−1^). Then, the culture was kept in the LD cycle (D) or exposed to a high level of light (500 μmol m^−2^ s^−1^) for 2 h (HL) with or without addition of DCMU or TEMPOL at h 12 (the onset of the second light period was defined as h 0). (B) MRE11-HA cells were synchronized by the low-light LD cycle. Then, the culture was kept in the LD cycle (D) or transferred to continuous low-light conditions with (L+DCMU) or without (L) addition of DCMU at h 12 (the onset of the second light period was defined as h 0). Images obtained by immunofluorescence microscopy show MRE11-HA protein localization at h 14 (A and B). Green, MRE11-HA detected with the HA antibody; magenta, autofluorescence of the chloroplast. The percentage of cells that exhibited MRE11 foci in the nuclei (examples are indicated with arrowheads in microscopic images) in total cells is indicated in the graph. Each data point represents means ± SD of results from three biological replicates (*n *>* *200 cells for each sample). Asterisks denote statistically significant differences (*, *P < *0.05; **, *P < *0.01 [*t* test]). Scale bars = 5 μm.

To examine whether the photosynthetic oxidative stress that occurred because of high levels of light exposure during the S-phase resulted in raised levels of DSB, either DCMU or 4-hydroxy-2,2,6,6-tetramethylpiperidine 1-oxyl free radical (TEMPOL; a ROS scavenger) was added to the culture immediately before the shift to high light at h 12 (Fig. 7A). The addition of DCMU or TEMPOL reduced the frequency of cells with MRE11 foci to a level similar to that seen without exposure to high levels of light (LD) (Fig. 7A). These results suggest that the frequency of DSB was increased by high levels of light exposure during the S phase because of photosynthetic oxidative stress.

Even under conditions of low light, the incidence of cells containing MRE11 foci under conditions of continuous light was more than two times that found under LD conditions at h 14 (when most of the cells were in the S phase) (Fig. 7B). When DCMU was added to the culture under continuous light at h 12, the incidence of cells containing MRE11 foci was reduced to a level similar to that found under LD conditions (Fig. 7B). These results suggest that nuclear DNA replication during photosynthesis increases the frequency of DSB in C. merolae.

Finally, we tested this conclusion by examining the effect of S-phase progression on the frequency of DSB during the daytime by depletion of RBR (Fig. S8). To this end, wild-type and Δ*RBR* cells expressing MRE11-HA were cultured under LD conditions (Fig. S8). The incidence of cells containing MRE11 foci was higher in Δ*RBR* cells than in the wild-type cells at h 6 (light period; Fig. S8). In addition, the incidence of cells containing MRE11 foci was higher at h 6 than at h 18 in Δ*RBR* cells. This was in contrast to the wild-type cells, in which the frequency of MRE11 foci was higher at h 18, which corresponds to the S and M phases, than at h 6 (Fig. S8). This result suggests that the G_1_/S transition that occurs during daytime (in Δ*RBR* strains) increases the rate of DSB and thus supports the conclusion presented above.

10.1128/mBio.00833-19.8 FIG S8 The effect of daytime progression of the S phase on the frequency of nuclear DSB. Wild-type (WT) and Δ*RBR* cells expressing MRE11-HA were synchronized by the LD cycle (100 μmol m^−2^ s^−1^). Cells were harvested at h 6 (during the second light period) and h 18 (during the second dark period); they were subsequently immunostained with the anti-HA antibody. Green, MRE11-HA detected with the HA antibody; magenta, autofluorescence of the chloroplast. The percentage of cells that exhibited MRE11 foci in the nuclei (examples are indicated with arrowheads in microscopic images) is indicated in the graph. Each data point represents means ± SD of results from three biological replicates (*n *>* *200 cells for each sample). Asterisks denote statistically significant differences (*t* test; *, *P < *0.05; **, *P < *0.01). Scale bars = 5 μm. Note that Δ*RBR* cells are smaller than wild-type cells (18). WT and Δ*RBR* cells expressing MRE11-HA was diluted with a fresh medium to give a concentration of OD_750_ = 0.2 (∼0.6 nmol chlorophyll *a*/ml culture), before the cells were subjected to the LD cycle. Download FIG S8, PDF file, 0.7 MB. Copyright © 2019 Miyagishima et al. 2019 Miyagishima et al. This content is distributed under the terms of the Creative Commons Attribution 4.0 International license.

## DISCUSSION

**FIG 8:**
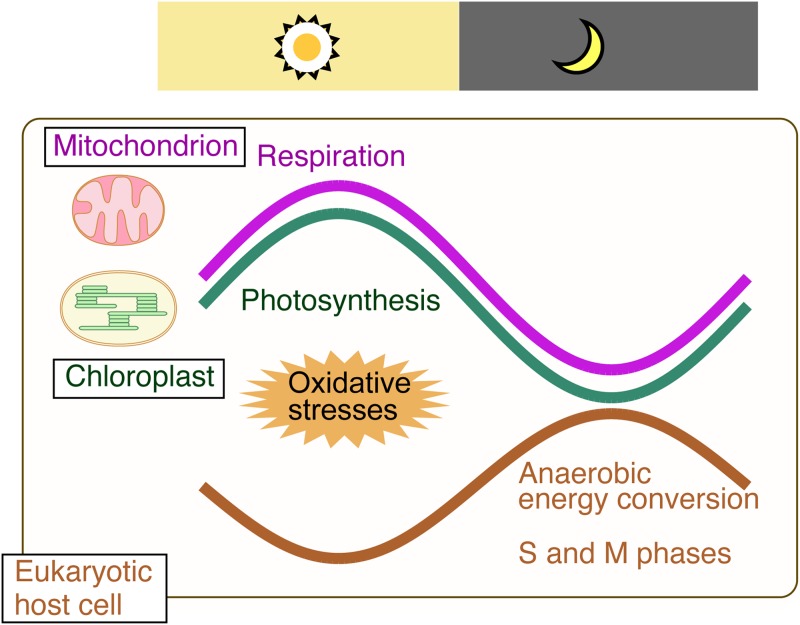
Schematic representation of the relationships between daily rhythms in respiratory and photosynthetic activities and cell cycle progression in the red alga C. merolae. Photosynthetic activity and respiratory activity peak in the morning and then decrease. In contrast, pathways for anaerobic pyruvate consumption are upregulated during the evening and night. The temporal separation of ROS-generating oxygenic energy metabolism by mitochondria and chloroplasts from nuclear DNA replication ensures safe cell proliferation.

We have shown that, in C. merolae, photosynthetic and respiratory activities are downregulated in the evening and at night when cells enter the S phase. In addition, our results suggest that this temporal sequestration of oxygenic energy conversion and G_1_/S transition reduces the risk of nuclear DSB (Fig. 8).

In C. merolae, respiratory activity was lower during the night than during the day whereas our transcriptome and metabolome data suggest that more pyruvate is anaerobically consumed to continue ATP production by glycolysis (Fig. 2 and Fig. 3; see also Fig. 8). A plausible explanation for this difference between the results determined during the day and night is as follows. During the day, when biomass is increasing, there is demand for TCA cycle intermediates and NAD(P)H for synthesis of biomolecules in addition to ATP. In contrast, in the night, the increase of biomass ceases and thus, the demand for TCA cycle intermediates and NAD(P)H decreases whereas ATP is still required for nuclear DNA replication and segregation and organelle and cell division.

A very recent study in C. reinhardtii which was published during preparation of this paper also showed that the level of respiratory activity was lower during the night than during the day whereas genes and proteins related to multiple fermentation pathways are upregulated during the night (24). Consistent with these observations, it was shown that lactate accumulates during the evening and night (24). These results suggest that pyruvate is metabolized anaerobically rather than by TCA cycle during the night in C. reinhardtii (24) as suggested by this study in C. merolae. Related to this point, we examined the published transcriptome data of C. reinhardtii cultured under LD conditions (23). The result showed that, in addition to the pyruvate dehydrogenase E3 component, which was downregulated also in C. merolae, the E1 alpha, E1 beta, and E2 components were transcriptionally downregulated during the evening and night (see Fig. S9 in the supplemental material), in contrast to mRNAs encoding enzymes involved in fermentation pathways (PFL1, ADH, PAT1/2, ACK1/2, and LDH) (35, 36), which were upregulated in the evening in C. reinhardtii as shown recently (24) (Fig. S9).

10.1128/mBio.00833-19.9 FIG S9 Temporal changes in levels of mRNAs that encode components of the pyruvate dehydrogenase complex and fermentation enzymes in the green alga C. reinhardtii. Data correspond to results of transcriptome sequencing (RNA-seq) analyses performed by Zones et al. (23), in which C. reinhardtii cells were synchronized under conditions of an LD cycle in an inorganic photoautotrophic medium. Download FIG S9, PDF file, 0.5 MB. Copyright © 2019 Miyagishima et al. 2019 Miyagishima et al. This content is distributed under the terms of the Creative Commons Attribution 4.0 International license.

As in C. merolae, C. reinhardtii enters S phase during evening and early at night (11, 17, 23, 24). However, in terms of the exact timing in the laboratory culture under LD conditions, nuclear DNA synthesis starts in the light before the onset of the dark period (11, 23, 24). A recent transcriptome analysis showed that the level of mRNAs of genes involved in chromosome replication peaks at h 11 in the light period (24). However, in terms of natural environment, these observations indicate that the cells enter S phase in evening and hence that the light intensity becomes very low in nature. Related to the issue of the natural environment, C. merolae and its relatives (cyanidialean red algae) inhabit acidic volcanic hot springs, where they grow at the bottom of shallow hot pools (∼5 cm in depth) and on rocks exposed to fumes above the water surface (52). Around noon in summer, they are exposed to a much higher level of light (1,000 to 2,000 μmol m^−2^ s^−1^) than cultures that has been used in this study. Thus, in natural habitats, the temporal separation of photosynthesis and nuclear DNA replication would have a higher impact on “safe” proliferation than that suggested by results of this study.

Many algae reproduce asexually at the haploid stage, with several species maintaining haploidy throughout their life cycles, as in the case of C. merolae (53). In a haploid cell, DSB repair by homologous recombination is impossible before the region has been replicated during the S phase and mutations are introduced at a higher frequency by repair with nonhomologous end joining. Thus, the temporal separation of oxygenic energy metabolism and nuclear DNA replication is likely to aid the survival of certain algal species.

In the budding yeast Saccharomyces cerevisiae, under nutrient-limiting conditions, cells rhythmically alternate the balance of glycolysis and respiration. Glycolysis (reductive phase) and respiration (oxidative phase) are upregulated alternately over a cycle of 4 to 5 h, and the G_1_/S transition is restricted to the reductive phase (50, 54). In addition, it has been shown that this temporal restriction of the S phase reduces mutations in the genome (54). In some mammalian cells, it has been demonstrated that respiratory activity and G_1_/S transition are regulated by a circadian rhythm at the transcriptional level. Respiratory activity is upregulated during the day, whereas the G_1_/S transition occurs during the night (55–57). Thus, it is plausible that several lineages of nonphotosynthetic eukaryotes temporally separate mitochondrial respiration and nuclear DNA replication to allow safe replication of nuclear DNA and cells. However, the situation is more complex in photosynthetic eukaryotes because, in addition to respiration in the mitochondria, photosynthesis in chloroplasts generates ROS at what is believed to be much higher levels than those produced by respiration in mitochondria (3).

Nuclear DNA replication during the evening and night has been observed in several algal species (11–20), but it should be noted that this is not the only way to solve the toxic effect of oxidative stress on DNA replication. Some algae are known to divide predominantly during the day (58). However, it is noteworthy that the timing of cytokinesis is based on an increase in cell number in many studies. As such, it is still possible that nuclear DNA replication occurs during the night in some cases. Seed plants seemingly developed spatial rather than temporal separation of energy-generating processes and DNA replication, by which cell division was restricted mainly to nonphotosynthetic meristematic tissues (5). Under photoautotrophic conditions, DNA replication in cyanobacteria and chloroplasts occurs during the day, depending on the electron flow through the photosystems (59, 60). Both cyanobacteria and chloroplasts possess multiple copies of the chromosome per cell or organelle. In cyanobacteria, nucleoids are surrounded by many layers of thylakoid membrane, which probably functions to reduce the intensity of light reaching the photosystems that are close to the DNA. In addition, multiple copies of the chromosome are replicated asynchronously in individual cells (61, 62). If a chromosome is damaged during replication, the other copies of the chromosome are likely to function as reserves (62). In summary, there are several means of protecting the DNA replication process from the oxidative stress caused by oxygenic energy metabolism. This is similar to nitrogen fixation in cyanobacteria, where nitrogenases are irreversibly inhibited by dioxygen. Some species spatially separate oxygen-producing photosynthesis from nitrogen fixation by expressing nitrogenases in specialized nonphotosynthetic anaerobic cells called heterocysts (63). Other species solve the incompatibility of the processes temporally by regulating nitrogenase activity with a day/night or circadian rhythm and maximizing the activity during the night, when oxygen-producing photosynthesis does not operate (63).

In this study, we found that the temporal separation of oxygenic energy conversion and nuclear DNA replication is important for reducing the risk of DSB in eukaryotic algae. However, there are probably activities of the eukaryotic host cell, in addition to nuclear DNA replication, that are sensitive to and/or incompatible with oxygenic energy metabolism by endosymbiotic organelles. Further studies on the strategies adopted by eukaryotes to solve these issues will provide important insights into how eukaryotic cells coordinate the activities of the host cell and endosymbiotic organelles.

## MATERIALS AND METHODS

### Algal culture.

C. merolae 10D and its derivatives were grown in 2× Allen's medium (an inorganic autotrophic medium) (52). For C. merolae synchronization, the cells were cultured in 700-ml flat bottles (600 ml culture medium; 60 mm thick) and subjected to a 12-h light/12-h dark cycle (white fluorescent lamps, 5,000 K; 100 μmol m^−2^ s^−1^, unless otherwise indicated) at 42°C under aeration with ordinary air (3 liter min^−1^). For treatment with DCMU, 120 μl of 50 mM DCMU stock solution–ethanol was added to the 600-ml culture to give a final concentration of 10 μM. For treatment with TEMPOL, a 1/500 volume of 1 M TEMPOL stock solution in water was added to the culture to give a final concentration of 2 mM.

To measure oxygen consumption rate or evolution rate and for metabolome analyses, quantitative RT-PCR, and immunoblot analyses, a stationary culture was diluted with a fresh medium to give a concentration of optical density at 750 nm (OD_750_) of 0.2. Then, the cells were cultured under continuous light for 2 days until the OD_750_ reached ∼0.6. Then, cells were cultured in the dark for 24 h and then subjected to the 12-h light/12-h dark (LD) cycle. For microarray analyses, the stationary culture was diluted with fresh medium to give a concentration of OD_750_ of 0.2 before the cells were subjected to the LD cycle. Synchrony of the respective C. merolae cultures described above was confirmed by the predominant existence of dividing cells in the dark period revealed by microscopic observation.

The unicellular green alga C. reinhardtii 137c mt^+^ (CC-125) was grown in Sueoka’s high-salt medium (HSM; an inorganic autotrophic medium) (64). For C. reinhardtii synchronization, cells were diluted with 600 ml of the fresh medium to give a concentration of OD_750_ of 0.002 in 700-ml flat bottles and were cultured under conditions of continuous light (100 μmol m^−2^ s^−1^) for 3 days. Then, the cells were cultured in the dark for 24 h and subjected to a 12-h light (white fluorescent lamps as described above; 100 μmol m^−2^ s^−1^)/12-h dark LD cycle at 24°C under conditions of aeration with ordinary air (1 liter min^−1^). Synchrony of the C. reinhardtii culture was confirmed by the detection of dividing cells specifically in the dark period by microscopic observation.

### Measurement of oxygen consumption and evolution rates.

Oxygen consumption and evolution rates were measured using an oxygen electrode (Oxytherm system composed of an S1/MINI Clark type electrode disc and OXYT1 electrode control unit; Hansatech, King’s Lynn, United Kingdom). A 2-ml culture was loaded into the electrode chamber. The rates in C. merolae were measured at 40°C and in C. reinhardtii at 24°C with stirring the culture at 100 rpm. To measure oxygen consumption by respiration, the chamber was kept in the dark. To measure oxygen evolution by photosynthesis, the electrode chamber was illuminated with the indicated light intensity. Oxygen consumption was determined without any external substrates. Where indicated, a 1/100 volume of 1 M NaHCO_3_ was added to the culture before measurement of oxygen evolution. The chlorophyll *a* concentration was determined using a previously described method (65).

### Microarray transcriptome analysis, CE-MS metabolome analysis, and quantitative RT-PCR.

RNA extraction and microarray analyses were performed using a customized oligonucleotide DNA microarray (Agilent Technologies, Santa Clara, CA, USA) (8 samples × 15,000 probes/chip, 4,947 probes × three technical replicates for one sample; the mean of results from three technical replicates is shown as the signal of a gene at given time point in Fig. 2) (see also Fig. S2 and Fig. S3 and Data Set S1 in the supplemental material), as described previously (66). A 50-ng aliquot of total RNA for each sample was examined with quantile normalization. The analysis described above was performed twice to evaluate the reproducibility of the results by using cells cultured under LD conditions at different times. CE-MS analyses were performed using a previously described method (66) for three independent LD cultures. Quantitative RT-PCR was performed with 1/500 aliquot of cDNA prepared from 1 μg total RNA for each sample using Power SYBR green PCR Master Mix (Applied Biosystems) as described previously (67) with the primers listed in Data Set S1. *DRP3* (CME019C) was used as the internal control.

### Preparation of the MRE11-HA-expressing C. merolae strain.

Primers used for the plasmid construction are listed in Data Set S1. The 3× HA epitope-coding sequence was inserted just before the stop codon of *MRE11* (CMB035C) *orf* in the C. merolae chromosome to produce a C-terminal 3× HA-tagged MRE11-expressing stable transformant by the *MRE11* promoter. The *MRE11* locus around the stop codon (∼1 kb 5′ and ∼1 kb 3′ genomic sequence) was amplified from genomic DNA with primers B035C_stop-1000f and B035C_stop+1000r and was cloned into pGEM-T Easy vector (Promega). The resultant plasmid was linearized by PCR amplification with primers B035C_vec_F and B035C_vec_R. The 3× HA-coding sequence linked to the beta-tubulin (CMN263C) 3′-untranslated region (0.2 kb) and the Cm*URA* transformation marker, which was amplified with primers HA_URA_F and HA_URA_R plus the pQED3HAnosUra vector (the *nos* terminator of the vector was replaced with the beta-tubulin 3′-untranslated region) used as the template, was cloned into the linearized vector by the use of an In-Fusion cloning kit to produce the *MRE11*_*HA*_*URA* plasmid. A 5-μg volume of the PCR product amplified from this plasmid by the primers B035C_stop-1000F and B035C_stop+1000R was used for the transformation of C. merolae, which was performed as described previously (52).

To produce strain Δ*RBR* expressing MRE11-HA, the *RBR* locus was replaced with a chloramphenicol resistance gene (*CAT*) in the MRE11-HA cell prepared as described above. The RBR *orf* gene (CMT038C) and its 3′- and 5′-flanking genomic sequences were amplified with primers RBR(−1481)Fpuc5 and RBR(+572)Rpuc3 and cloned into pUC19 vector. The vector was linearized by PCR with primers RB(−1)Rcat5 and RB(2000). *CAT* was amplified with primers CAT5F and CAT3R and conjugated with the linearized vector using an In-Fusion cloning kit. A 5-μg volume of the PCR product amplified from this plasmid by the primers RB(−1481)Fpuc5 and RB(+572)Rpuc3 was used for the transformation of MRE11-HA strain, which was performed as described previously (52).

### Antibodies and immunoblot analyses.

Immunoblotting was performed as described previously (18), using anti-C. merolae FtsZ2-1 antibody (1:10,000) (26), anti-C. reinhardtii FtsZ1 antibody (1:10,000) (26), anti-HA antibody(clone 16B12; BioLegend, San Diego, CA, USA) (1:1,000), anti-alpha-tubulin monoclonal antibody (B-5-1-2; Sigma) (1:1,000), or anti-H3S10p antibody (06-570; Millipore) (1:1,000) as the primary antibody.

### Immunofluorescence microscopy.

Immunofluorescence staining was performed using a previously described method (68). Anti-HA antibody (clone 16B12; BioLegend) (1:500) and Alexa Fluor 488 goat anti-mouse antibody (Life Technologies) (1:1,000) were used as the primary and secondary antibodies, respectively. The immunostained sample was mixed with the same volume of 1 μg/ml 4'6-diamidino-2-phenylindole (DAPI) to visualize the DNA. The cells were examined using a 100× lens objective and an epifluorescence microscope (BX51; Olympus).
