# Supplementary figures and images for: Day/Night Separation of Oxygenic Energy Metabolism and Nuclear DNA Replication in the Unicellular Red Alga Cyanidioschyzon merolae
Source: mBio. 2019 Jul 2;10(4):e00833-19. doi: 10.1128/mBio.00833-19 (PMC6606799; doi:10.1128/mBio.00833-19)

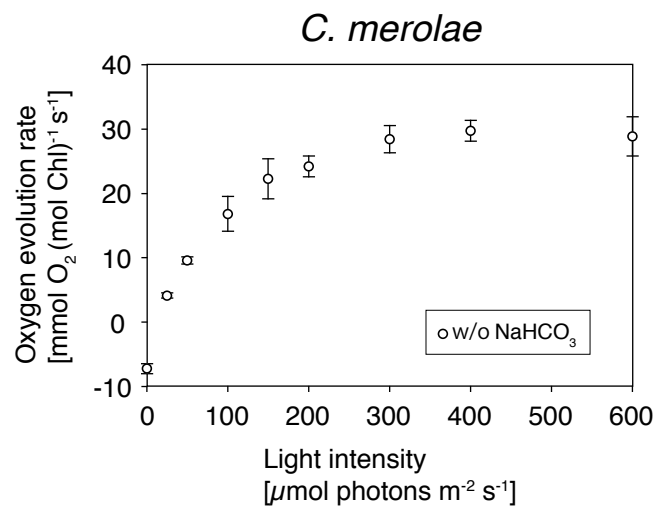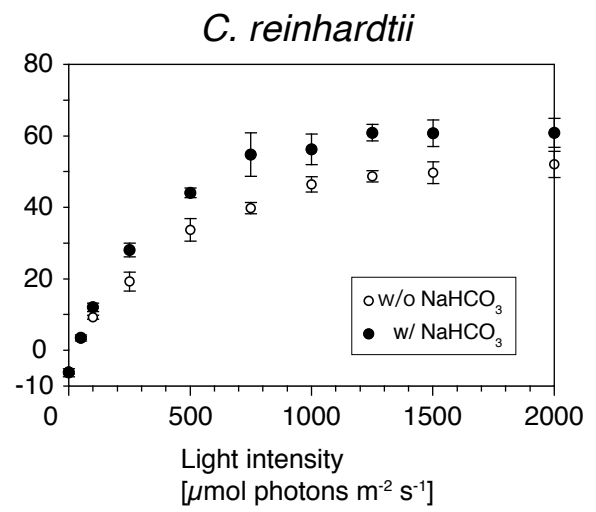

Supplement: FIG S1 [file mBio.00833-19-sf001.pdf]

Photosystems

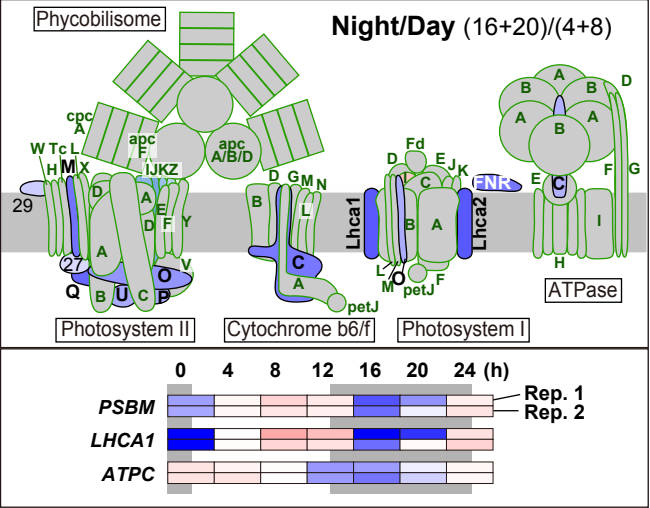

Calvin cycle

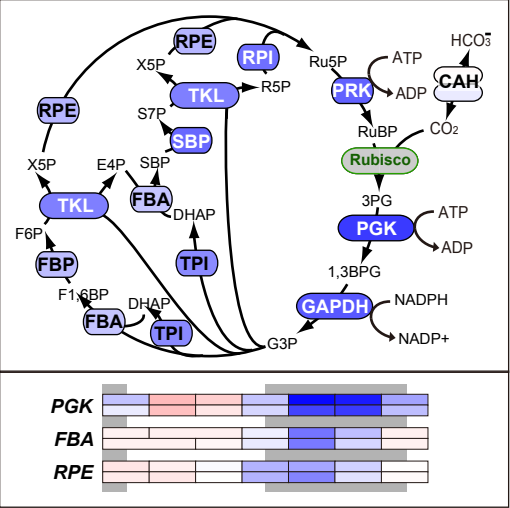

Respiratory chain

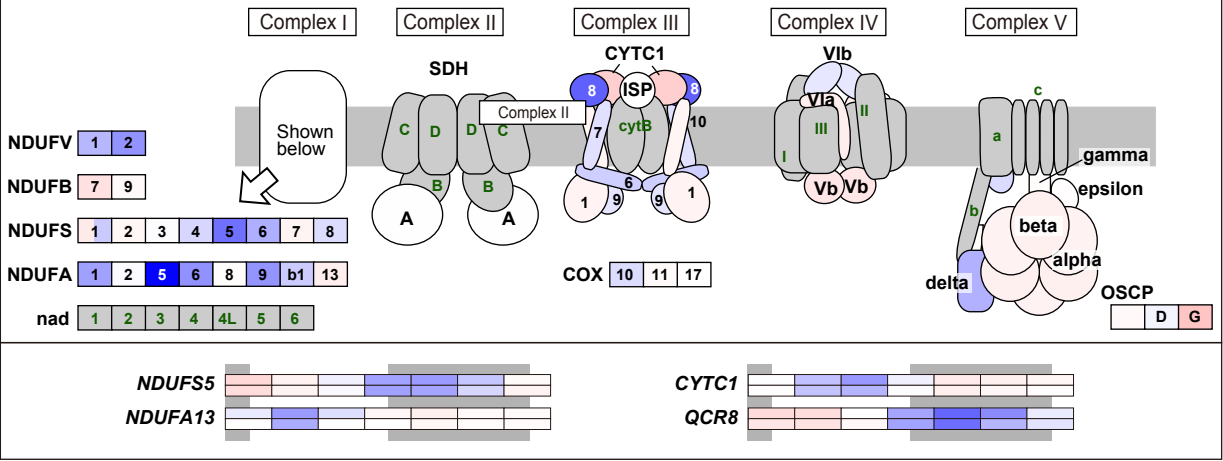

Cell cycle

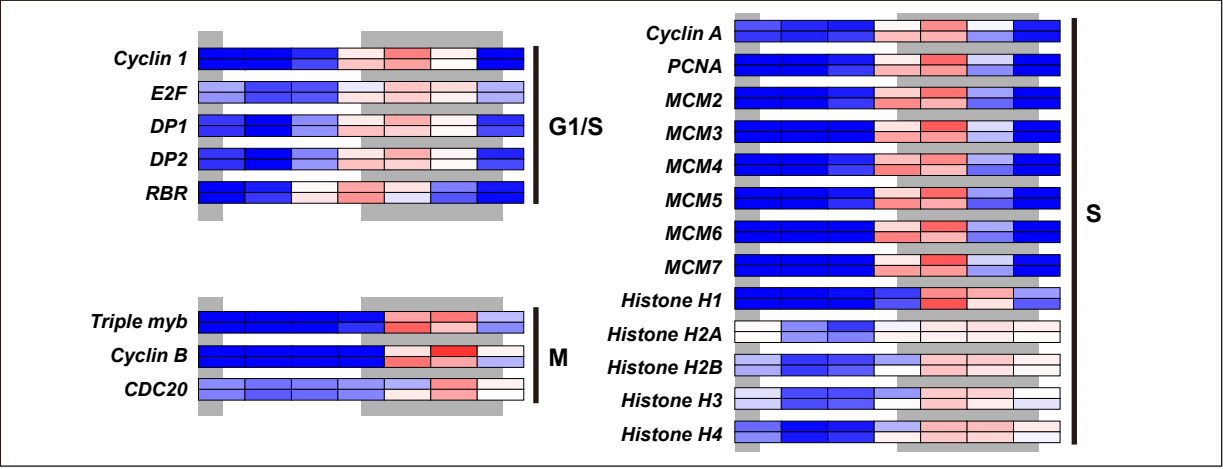

Supplement: FIG S2 [file mBio.00833-19-sf002.pdf]

Rep. 1

Rep. 2

### Pyruvate dehydrogenase complex

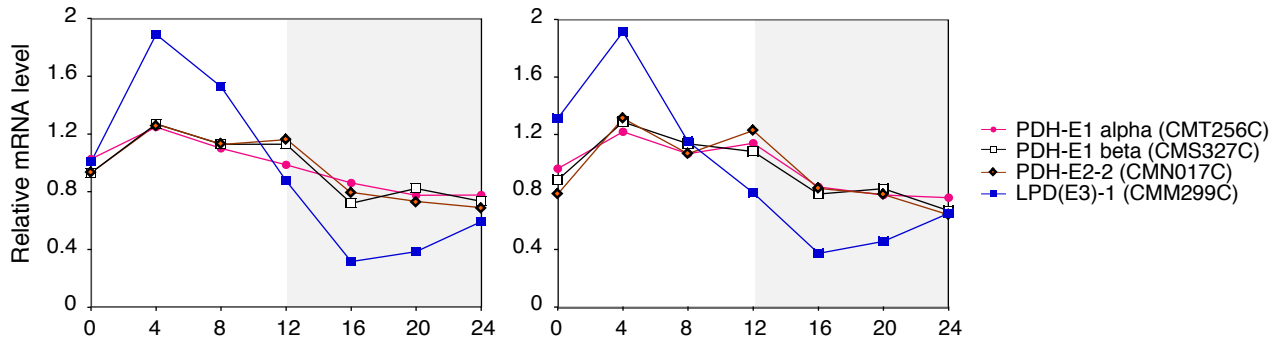

### Pyruvate kinases

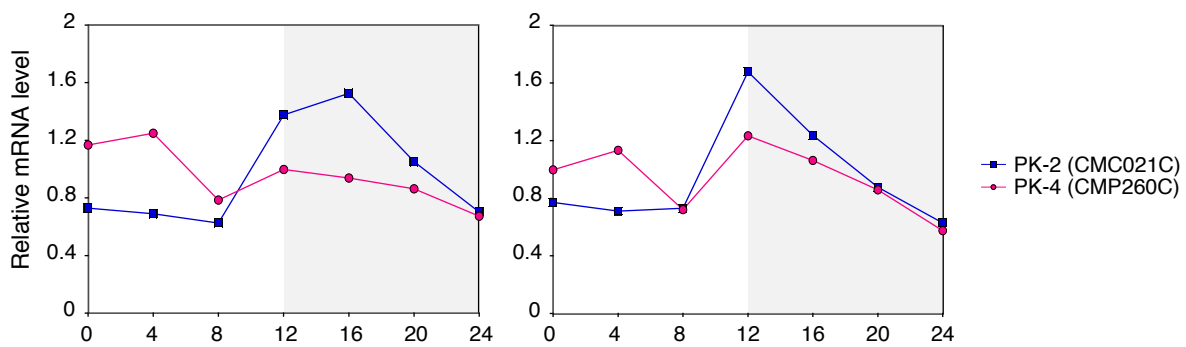

### TCA cycle enzymes

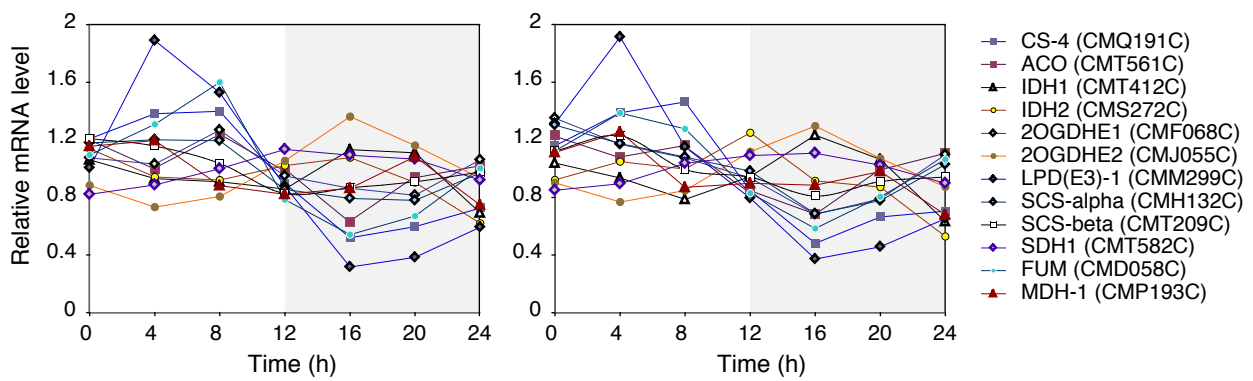

Supplement: FIG S3 [file mBio.00833-19-sf003.pdf]

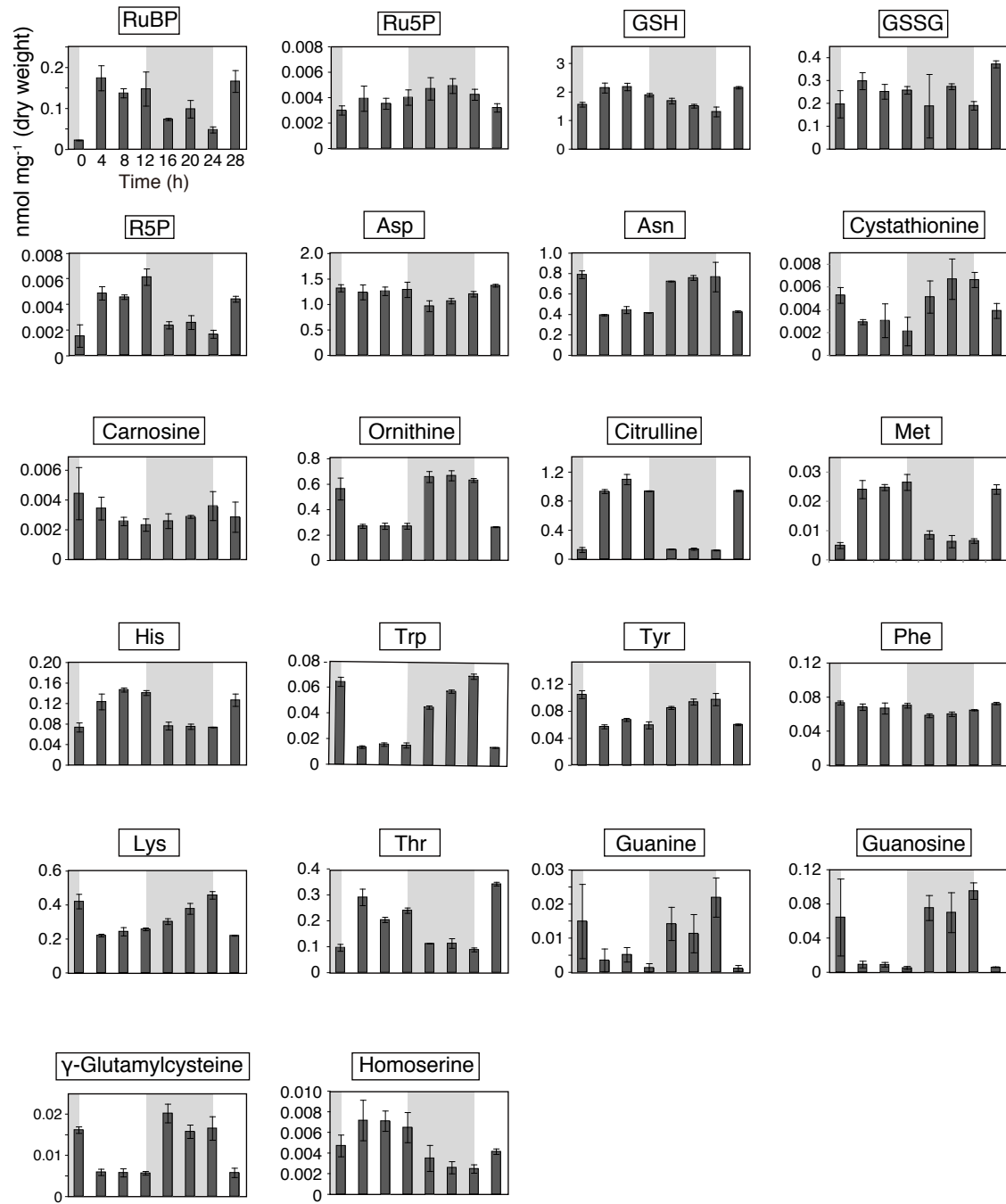

Supplement: FIG S4 [file mBio.00833-19-sf004.pdf]

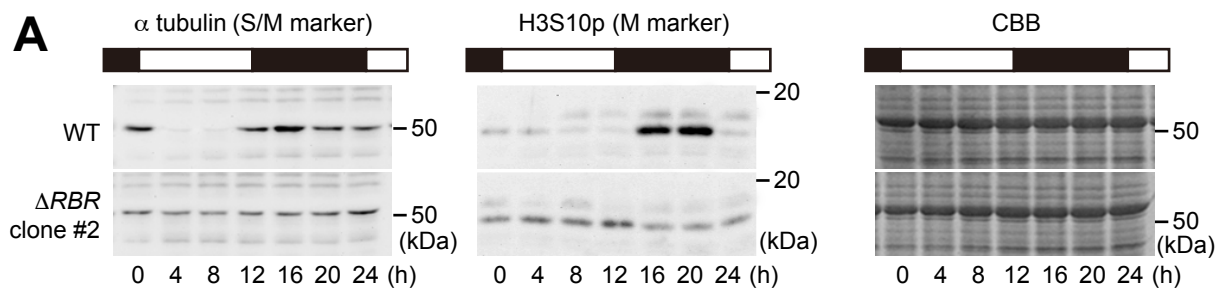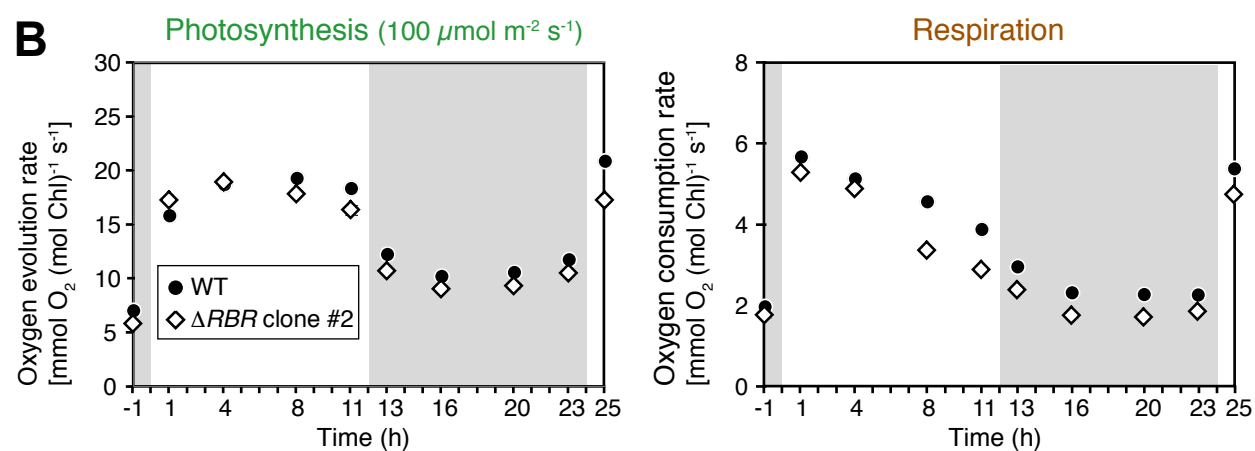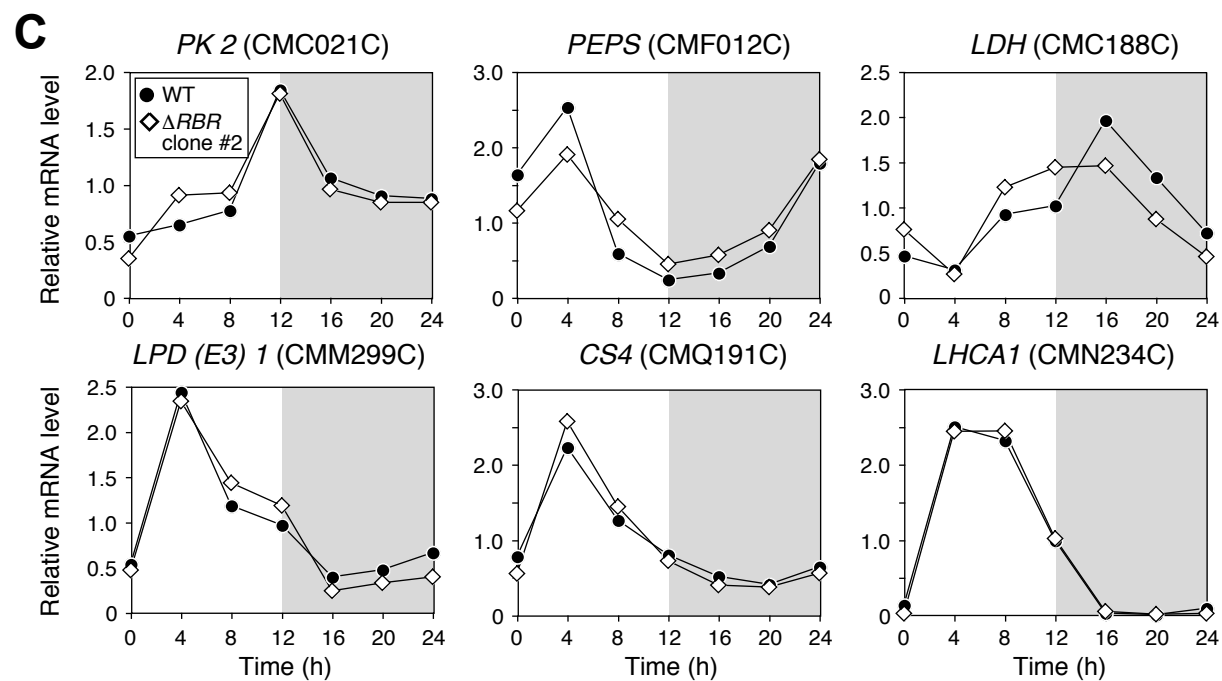

Supplement: FIG S5 [file mBio.00833-19-sf005.pdf]

Photosynthesis (Staurated light; 500  $\mu\text{mol m}^{-2} \text{s}^{-1}$ )

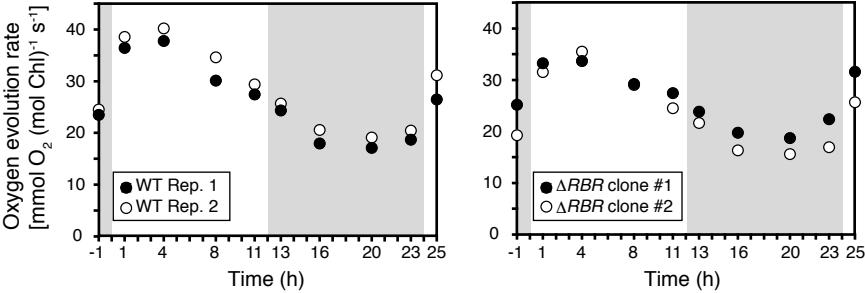

Supplement: FIG S6 [file mBio.00833-19-sf006.pdf]

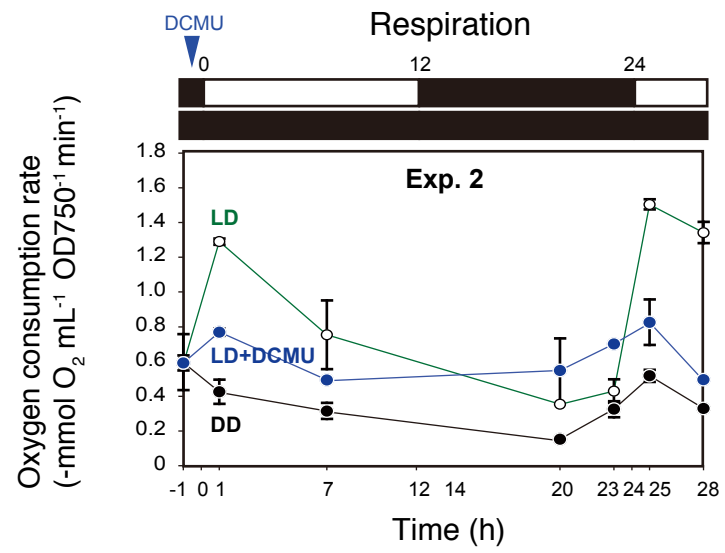

Supplement: FIG S7 [file mBio.00833-19-sf007.pdf]

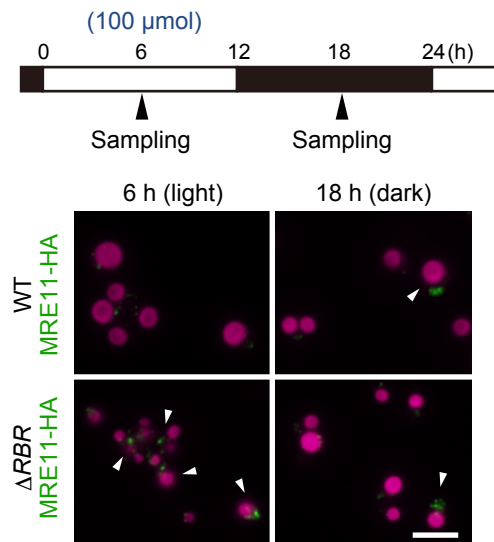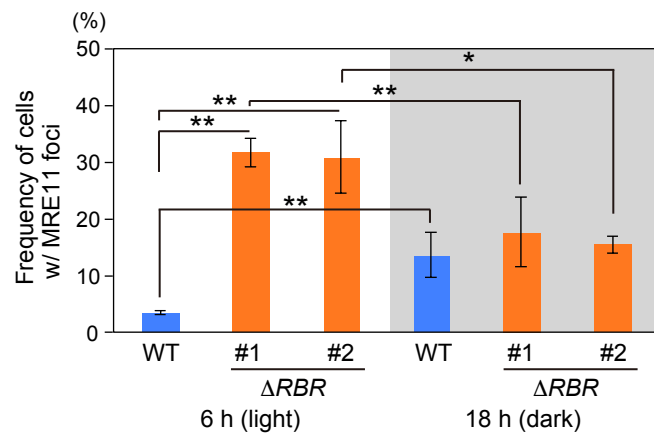

Supplement: FIG S8 [file mBio.00833-19-sf008.pdf]

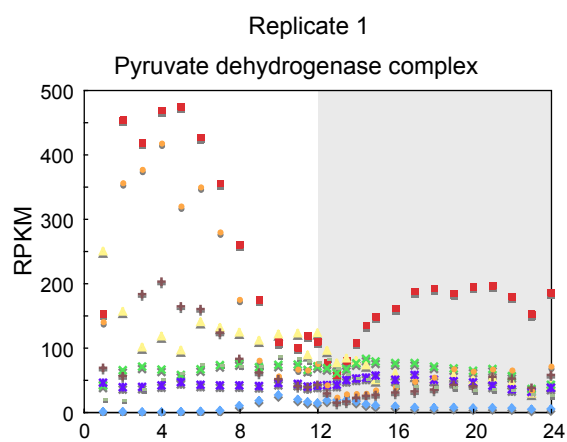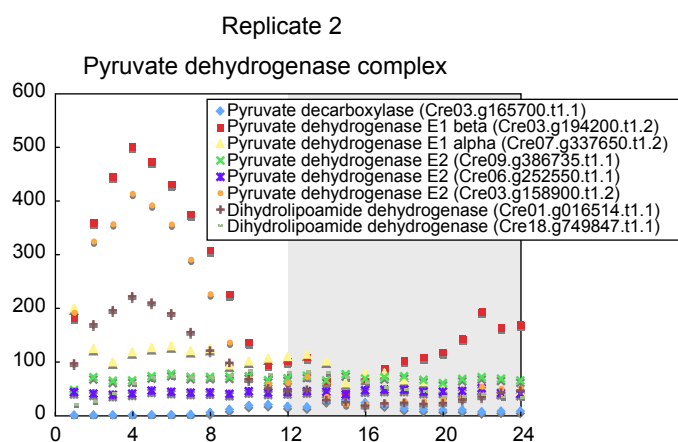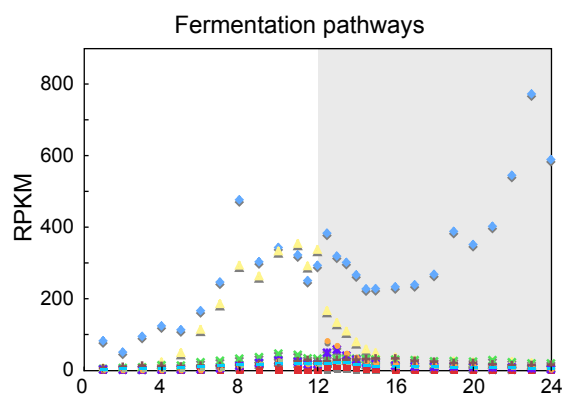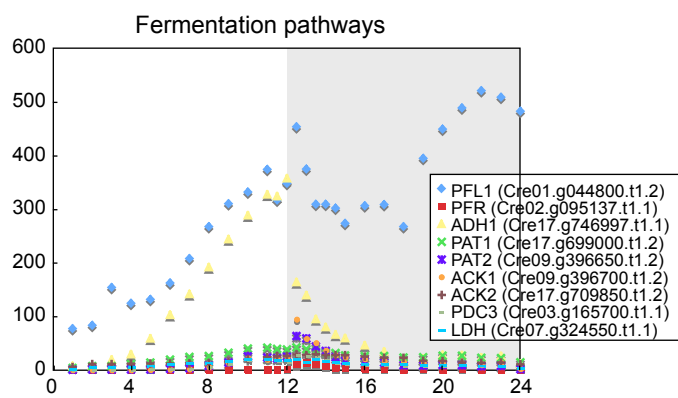

Supplement: FIG S9 [file mBio.00833-19-sf009.pdf]
